# Sexual dimorphism in the cell number of the adult *Drosophila* brain

**DOI:** 10.1371/journal.pone.0342456

**Published:** 2026-02-18

**Authors:** Narendra Pratap Singh, Susan T. Harbison

**Affiliations:** National Heart, Lung and Blood Institute, National Institutes of Health, Bethesda, Maryland, United States of America; ShanghaiTech University, CHINA

## Abstract

**Background:**

Sexual dimorphism in animal behavior is common and may originate from differences in brain structure or function. In *Drosophila*, substantial male and female specific differences in gross brain anatomy, neuronal wiring, and behavior have been observed. However, it is not known whether global differences in the brain anatomy are strictly due to differences in neuronal patterning or if differences in cell number may also play a role.

**Principal findings:**

During optimization of the assay for transposase-accessible chromatin with sequencing (ATAC-seq) to analyze gene regulation in adult brain cells we observed a need for more brain tissue in males compared to females. This suggested that male brains might have fewer cells. To test this hypothesis, we isolated total nuclei from adult brains and counted them using a flow cytometer. We found that female fly brains have approximately 30% more cells than male brains. These differences in cell number also correlated with differences in the physical size of the brain.

**Conclusions:**

Our results suggest that male and female brains are not only differently wired but also have global differences in cell number that should be considered when analyzing differences in their behavior.

## 1. Introduction

The fruit fly *Drosophila melanogaster* is an ideal model system to study the genetic and neural basis of innate behaviors [1–7]. Studies have identified genetic pathways that underlie dimorphic sexual behaviors in *Drosophila* [6]. Anatomical analyses of adult *Drosophila* brains show gross sexual differences in critical regions of the brain: the mushroom body, the fan-shaped body, antennal lobe and olfactory glomeruli, and the lateral horn, which are required for essential life processes, including learning, memory, sleep, circadian rhythms, perception of environmental stimuli, and olfactory information processing [8,9]. This suggests that understanding the cause of the sexual dimorphism in the fly brain would be rewarding. Despite the rich history of *Drosophila* genetics, it has been challenging to estimate the total number of cells in the adult brain, and different studies have reported a range of 100,000–200,000 cells [10–14]. In a more recent study, authors counted brain cells using a hemocytometer and found that the adult brain has around 200,000 cells, with male and female brains having very similar numbers of cells [10]. However, this conflicts with gross anatomical analyses that reported larger brain regions in females [8].

In an effort to analyze gene regulation in adult brain cells of male and female *Drosophila* using ATAC-Seq, we noticed that the female brain may have more cells compared to the male. ATAC-seq employs an adapter-loaded transposon (Tn5) technology that preferentially inserts the adapter into more accessible regions of the genome, a process known as the tagmentation reaction [15–18]. These inserted adapter sequences facilitate PCR amplification and barcoding of open chromatin regions using primer sequences. Subsequent library sequencing and analyses enables genome-wide mapping of open chromatin regions that often overlap with gene-regulating regions of the genome. The ratio of adapter-loaded transposon to target DNA in the nucleus is critical to achieve an optimal tagmentation reaction. A typical tagmentation protocol for human cells is optimized for 50,000 cells but can be modified for the specific cell number and cell type required in a particular experiment [15,19]. During optimization of the ATAC-seq protocol for *Drosophila* brains, we found that we always needed greater amounts of male brain tissue than female to achieve optimal tagmentation for the same amount of adapter-loaded Tn5 transposase enzyme. This indicated that the female brain may have more cells compared to males. To further validate this observation and directly test it, we optimized a protocol to efficiently isolate nuclei from adult brains and then count them using a fluorescence-activated cell sorting (FACS) method using flow cytometry. Nuclei counting further confirmed that the female brain has about 30% more cells as compared to males.

## 2. Results

### A) Optimizing the tagmentation reaction for adult *Drosophila* brain tissue

We used Omni-ATAC-seq, an improved protocol designed to enhance permeabilization across various cell types by adding NP-40, Tween 20, and digitonin detergents [18]. These changes were observed to greatly increase the signal-to-background ratio and allowed for more universal application of the protocol to different tissue types. However, the tagmentation step of the ATAC-seq protocols requires optimization for a specific number of cells, different cell types and species, to ensure accurate quantification and comparison across multiple experiments. The critical cell-counting step requires additional instrumentation, slows down the process, and increases the exposure of the sample (nuclei) to *in vitro* conditions, which can reduce the quality of the ATAC-seq data [20]. To overcome these limitations, we replaced the cell counting step with tissue amount to optimize the tagmentation reaction for brain tissues of *Drosophila*.

**Fig 1:**
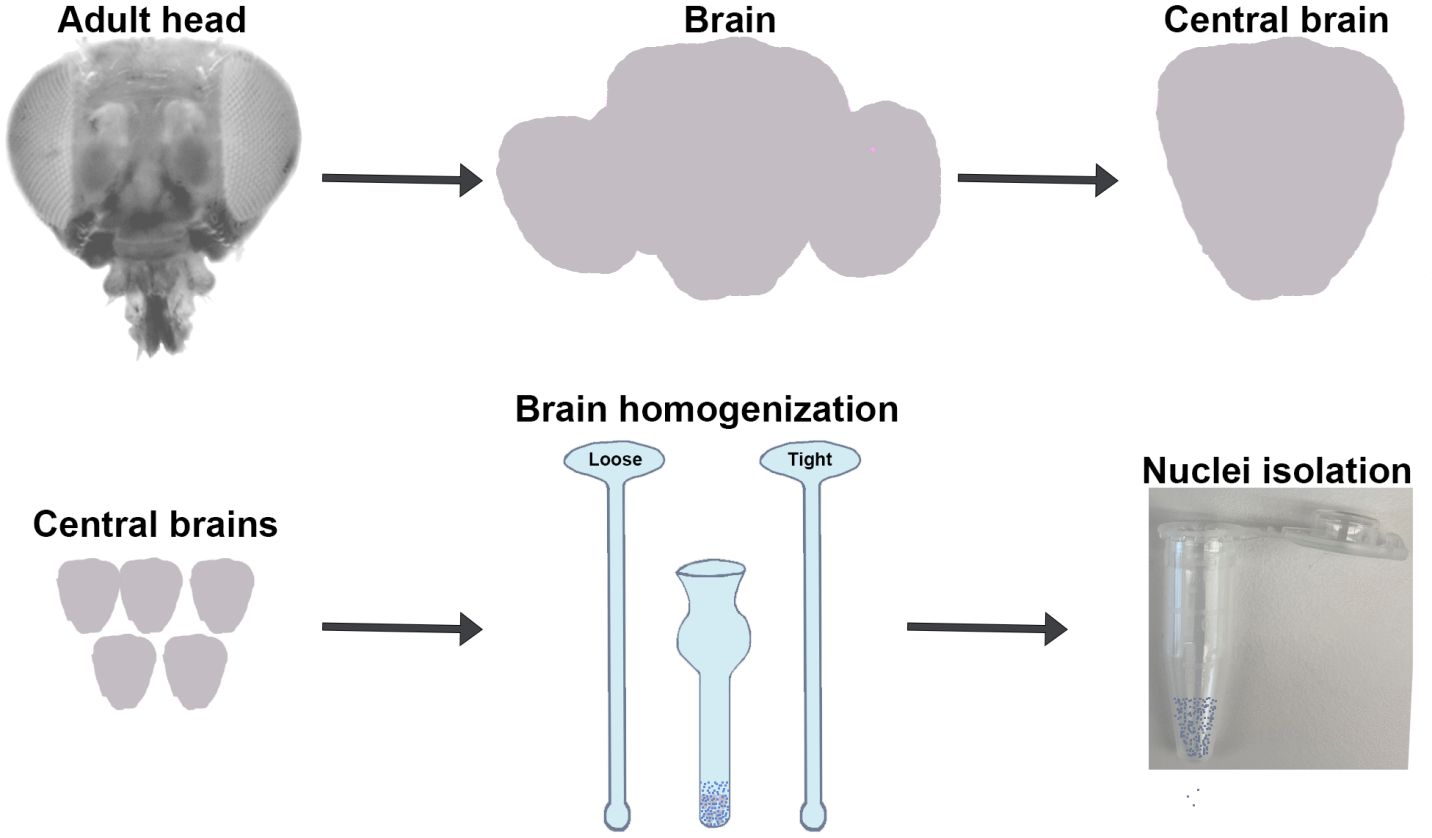
Isolation of central brain region: Schematic shows the isolation of the central brain from the head and homogenization to isolate nuclei.

**Fig 2:**
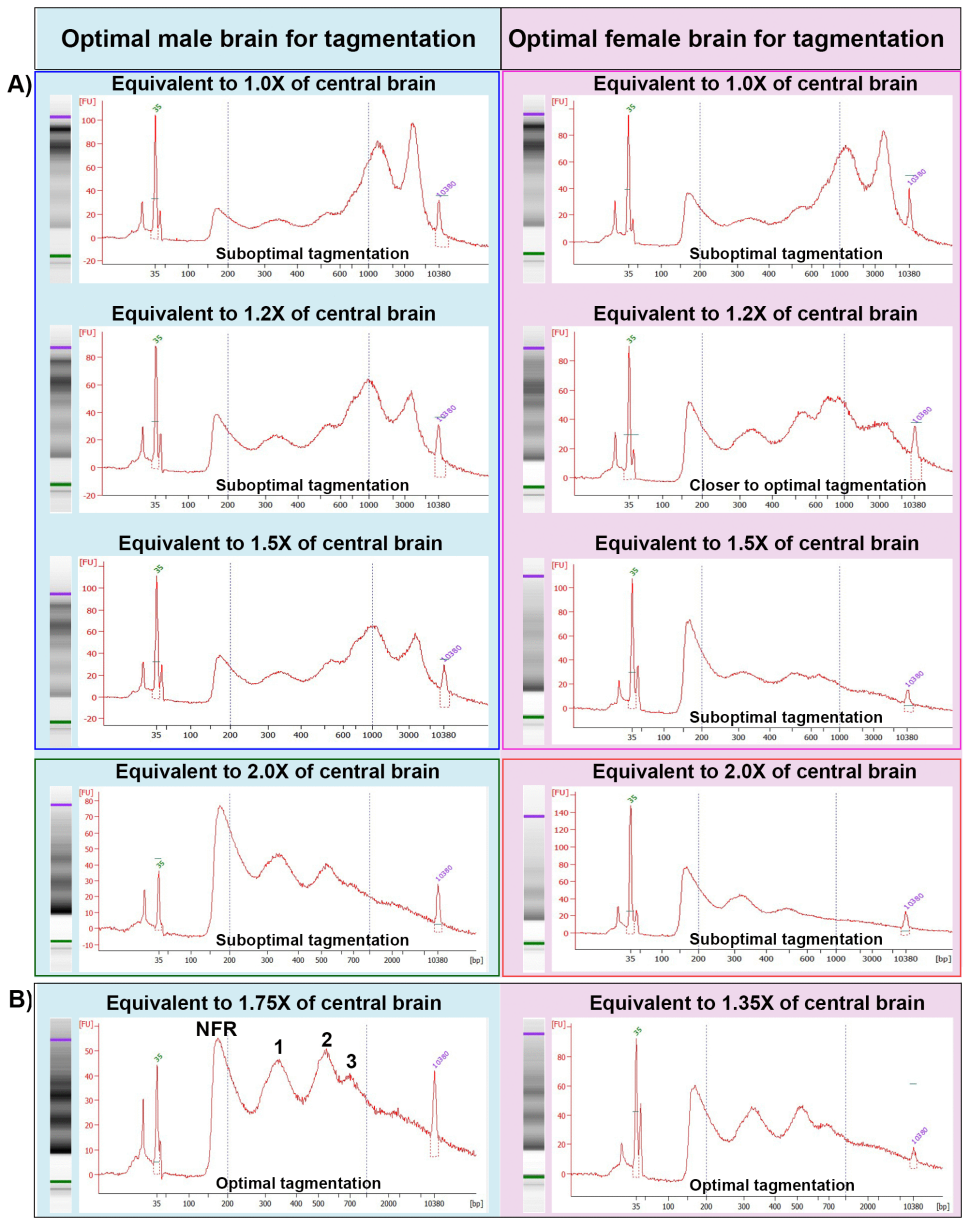
Bioanalyzer analyses of tagmented DNA: Bioanalyzer analysis of DNA fragments after tagmentation reaction is shown with the x-axis representing DNA size (base pairs), while the y-axis represents fluorescence intensity (concentration). Two vertical dotted lines on each picture represent 200 bp and 1kb size marker respectively. Experiments conducted on the same date are placed in the same box. A) DNA fragments after tagmentation reaction with nuclei equivalent to different fractions (1.0 ×, 1.2 ×, 1.5× and 2.0×) of central brain from male (blue), and female (pink) show suboptimal tagmentation. B) DNA fragments with nuclei of 1.75× of male 1.35× of female central brain show optimal tagmentation.

An optimal transposition reaction should produce ATAC-seq library fragments enriched with nucleosome-free regions (NFRs) with mono-, di-, and tri-nucleosomes, with sizes of approximately 180, 350, 540, and 700 base pairs, respectively [18,21], including a 135 bp adapter inserted by transposase. To optimize the transposition reaction, we dissected 5 central brains for each experiment and homogenized them to isolate the nuclei (Fig 1). At this step, we used Trypan blue stain to analyze brain dissociation and nuclei isolation. We observed high efficiency of nuclei isolation (>90%) and did not observe any difference in homogenization efficiency of male and female brains (S1 Fig). To control the nuclei number in the tagmentation reaction, we used variable volumes of nuclei suspension equivalent to different brain amounts with a fixed amount of transposase. We used nuclei equivalent to 1.0, 1.2, 1.5, 1.75, and 2.0 times the male and 1.0, 1.2, 1.35, 1.5, and 2.0 times the female central brain (Fig 2). We observed that the ATAC-seq library fragmentation pattern with a lower amount of nuclei (1.0 × male and female samples) showed a pattern where the NFR and mono-, di-, and tri-nucleosomes were smaller than the large nucleosome fragments (> 800 bp), indicating suboptimal tagmentation (Fig 2A, top panel). As the amount of brain tissue and the number of nuclei increased (1.2×, 1.5× and 2.0 of male and 1.0 and 1.2× of female), the amount of larger fragments decreased and the amount of smaller fragments increased (Fig 2A, middle and lower panels). The tagmentation reaction with female 1.35× and male 1.75× samples showed a pattern where the NFR and mono-, di-, and tri-nucleosome peaks were comparable to each other and highly enriched compared to larger nucleosome fragments (> 800 bp), indicating a pattern of optimal tagmentation (Figs 2B and S2). As the nuclei number further increased in the female 1.5× and male 2.0× samples, the NFR peak greatly increased compared to the mono-, di-, and tri-nucleosomes peak (Fig 2A) indicating suboptimal tagmentation. In general, for a given amount of enzyme, the addition of too few cells leads to “over tagmentation” that should produce a majority of small fragments while the addition of too many cells leads to “under tagmentation” that should produce a majority of large fragments [19]. However, the pattern of tagmented DNA we observed in our experiments shows a reverse pattern than what is typically seen with mammalian cells [19]. One possibility is an inaccuracy with the Bioanalyzer with high molecular weight DNA, a phenomenon previously noted [19]. An additional possibility is species and cell type specific differences in the open chromatin distribution in the cell.

These results clearly showed that nuclei coming from 1.35 × female and 1.75 × male central brain are optimal for tagmentation. However, this also implies that the number of nuclei in 1.35 × female is equivalent to 1.75 × male brain, suggesting that the female brain may have more cells than the male brain.

### B) Female brains are larger in size

**Fig 3:**
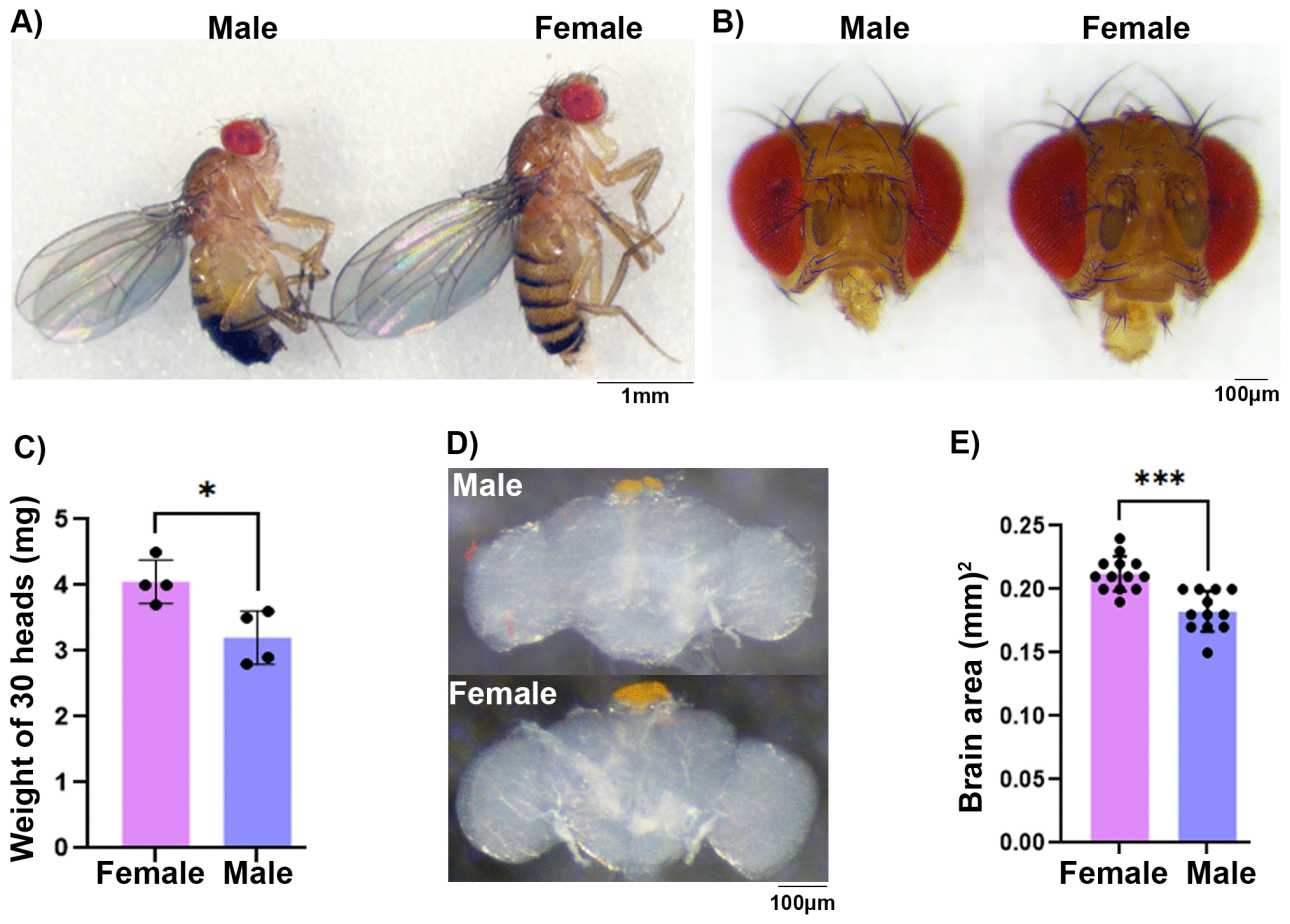
Analyses of brain size: A) Picture of adult male (left) and female (right) fly show differences in body size. B) Frontal view of the adult male (left) and female (right) head shows differences in the size. C) Quantification of head weight for 30 males and 30 females (two-way ANOVA, *P* value: 0.0179). D) Dissected adult male (upper) and female (lower) brain shows differences. E) Quantification of adult brain area shows larger brain size in females (two-way ANOVA, *P* value: < 0.0001).

To further investigate the sexual dimorphism in adult *Drosophila melanogaster* brain, we measured the whole body, adult head, and brain size. The *Drosophila* male body is known to be much smaller than female body, and this was also the case for the male head (Fig 3A and B; S1 and S2 Tables) [22,23]. To get quantitative differences in the head size, we analyzed the weight of an equal number of heads and observed that female heads were heavier than male heads (Fig 3C). On average, 30 female heads weighed 4.05 ± 0.16 mg, compared to 3.2 ± 0.20 mg for 30 male heads. Furthermore, we carefully dissected the brains from the adult heads and measured the area of whole male and female brains (Fig 3D). Quantitative analyses of brain area showed that, on average, female brains measured 0.2123 ± 0.0038 mm^2^, while male brains measured 0.1825 ± 0.0046 mm^2^ (Fig 3E). The larger brain size in females further supports the notion that they may have more cells than males.

### C) Female brains have more nuclei compared to males

**Fig 4:**
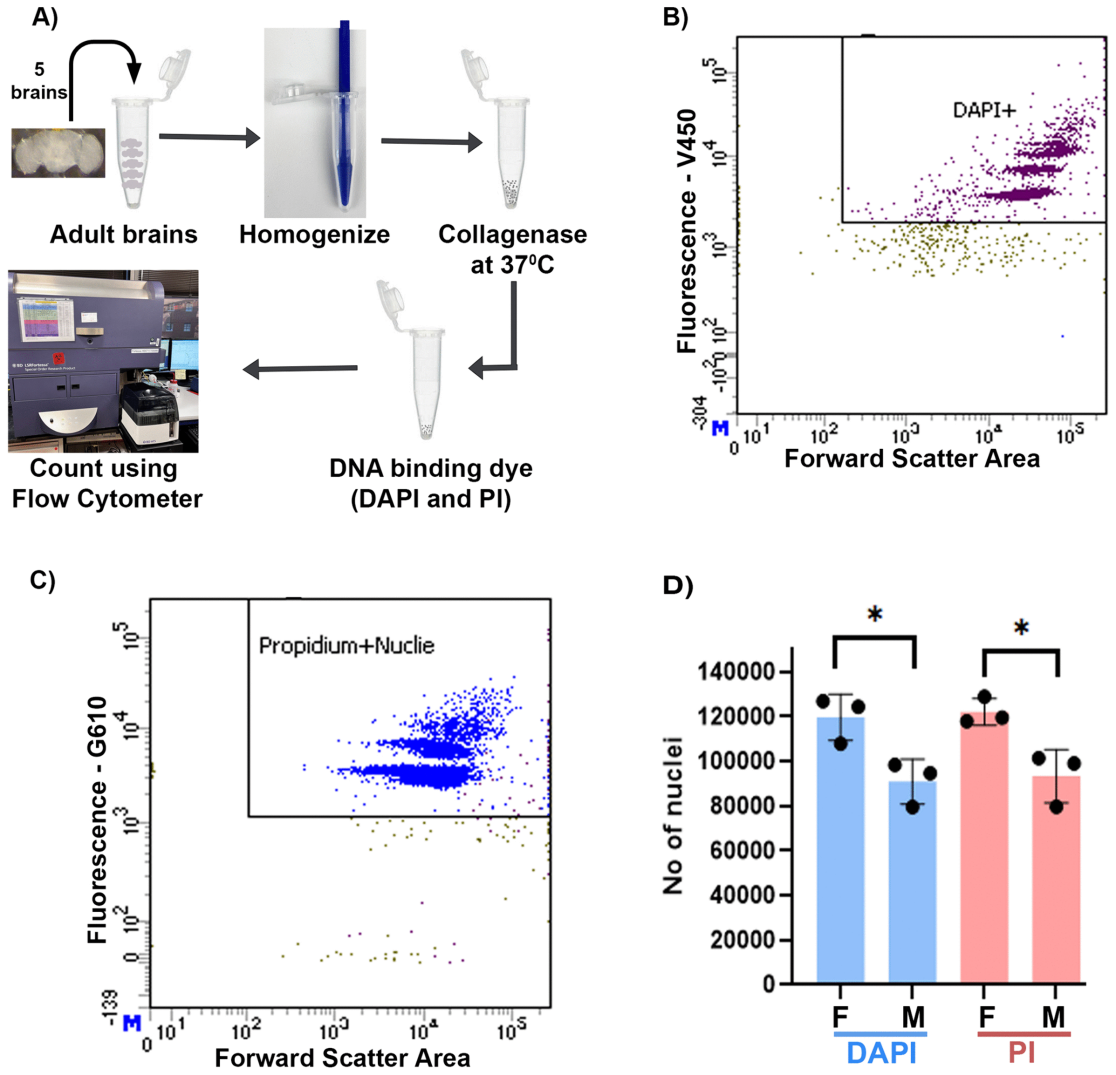
Analyses of brain nuclei number: A) Schematic shows nuclei isolation and counting using flow-cytometer from an adult *Drosophila* brain. B and C) Bivariate dot plot show sorting of nuclei using thresholds for size (Forward scatter area) and fluorescence DAPI and PI, respectively. D) Quantification of nuclei number in adult male and female central brain using DAPI and PI dyes shows that female has more nuclei (two-way ANOVA, *P* value: 0.0009).

To estimate the differences in brain cell number, we developed a protocol to isolate nuclei from the brain tissue and count the number of nuclei using fluorescence-activated cell sorting (FACS) with a flow cytometer (Fig 4A; S1 and S2 Tables). Since the brain hosts some of the largest cells (neurons) in the body, isolating intact cells and counting them individually is challenging. To overcome this limitation, we employed a physical and enzymatic disruption method to break the tissue and isolate nuclei for counting (Fig 4A). The use of the flow cytometer eliminated human biases and provided the ability to count larger numbers of nuclei for a more accurate estimate. Additionally, we used two dyes, Propidium Iodide (PI) and 4′,6-diamidino-2-phenylindole (DAPI), to stain the nuclei in FACS experiment in order to remove any dye biases (Fig 4B and C). We observed that more than 99% of nuclei isolated from brains were singlets in Flow cytometer counting, suggesting that the protocol worked efficiently to disrupt the brain tissue into a single-nuclei suspension (S3 Fig). Using this protocol, we found that female brains have an average of 121916 ± 3402 and 120521 ± 5771 nuclei based on the PI and DAPI staining, respectively (Fig 4D). In contrast, the male brain has an average of 93024 ± 6898 nuclei based on the PI staining and 91878 ± 5891 nuclei based on the DAPI staining. These results clearly show that the *Drosophila melanogaster* brain is sexually dimorphic in total nuclei number, with the female brain having roughly 30% more than the male brain. We did not observe a significant dye effect on nuclei number differences (S2 Table).

## 3. Discussion and conclusions

Animals have evolved a repertoire of sexually dimorphic innate behaviors that include mating, nursing, aggression, and territorial marking. These behaviors can be observed in naive animals without prior learning or experience, indicating that the neural circuits that mediate these behaviors must be developmentally programmed in the brain. Some of the sexually dimorphic innate behaviors have been mapped to the differential patterning of the nervous system in worms, flies, rats and mice [24]. In mice, the bed nucleus of the stria terminalis and the medial amygdala in the forebrain are larger in males, while the anteroventral periventricular nucleus and the dorsal and central linear raphe regions are larger in females, reflecting sexual dimorphism [24–27]. Similarly, in Drosophila, male brains show larger sexually dimorphic regions including the DA1 glomeruli of the antennal lobe, the pars intercerebralis, the medial tips of the mushroom body γ lobes, and the protocerebral arch, ring, and junction [8,28], while the mushroom bodies, fan-shaped body, antennal lobes, and lateral horn regions are larger in females, indicating sex-specific dimorphism [8,28].

Past studies have identified a large number of genes that regulate body and brain size in *Drosophila*, indicating that the genetic architecture underlying the regulation of size is complex with different genes contributing to various traits in a sexually dimorphic manner [23,28–35]. Most of these genes are related to development and metabolic functions [35]. The large scale sexually dimorphic gene expression in flies is believed to be driven by presence of two *X*-chromosomes in females as compared to one in males [1,36]. For example, the presence of two *X* chromosomes in females leads to the production of a functional Sex-lethal (SXL) protein, which regulates the female-specific splicing of the *transformer* (*tra*) gene [37]. The female specific TRA protein, in turn, directs the splicing of *doublesex* (*dsx*) and *fruitless* (*fru*) mRNAs into their female-specific isoforms. Conversely, in males, the absence of functional SXL and TRA-F proteins leads to the default splicing of *dsx* and *fru* mRNAs into male-specific isoforms and these proteins drive male development [1]. The *dsx* and *fru* genes code for transcription factors that have sexually dimorphic gene expression that determines sex specific neuronal patterning and cell number [38,39]. For example, male-specific P1 neurons regulate intermale aggression and courtship behavior while descending neurons coordinate courtship behaviors [40,41]. Similarly, female-specific oviposition descending neurons regulate egg laying and vaginal plate opening descending neurons regulate vaginal plate opening during copulation [42–44].

In this study, we used two independent methods, tagmentation and flow cytometer nuclei counting, to quantitate sex-specific cell number differences in the adult brain of *Drosophila*. Using the tagmentation reaction, where Tn5 transposase inserts an adapter sequence into the target genomic DNA, we found that the tissue required for optimal tagmentation in females is 1.35× of the central brain, while for males it is 1.75× of the central brain. This suggests that males have a lower amount of target DNA in each brain. The requirement of a higher amount of male brain tissue (around 30%) compared to females may be attributed to lower number of cells in the male brain [8,28]. To test if the need of a higher number of male brain cells in tagmentation reaction is due to a lower number of cells, we optimized an efficient collagenase-based nuclei isolation and flow cytometer-based counting protocol to directly count nuclei [45–47]. We isolated nuclei as a proxy and counted them to estimate the total cell number because isolation of intact neuronal cells from the brain is impractical due to their large cell size. For efficient nuclei isolation, we used homogenization and collagenase in combination with multiple detergents (refer to Materials and Methods) to rupture the cell membrane. To avoid loss of the nuclei, we minimized nuclei stickiness by adding 2% BSA, minimized the pipetting steps, and avoided centrifugation of the samples. For nuclei counting, we used a flow cytometer instead of the more common method of using a hemocytometer, which is time-consuming and prone to large technical variations due to human errors and sample preparation challenges [45,48,49]. Nuclei counting results showed that the female brain has around 120K nuclei, while the male brain has around 90K. The differences in cell number also correlate with differences in brain size, where the female brain is heavier and larger compared to that of the male. We speculate that these differences in the cell number are contributed by sex-specific enlarged regions, where male enlarged regions do not contribute enough cells to compensate for female enlarged regions [8,28]. These observations together suggest that male and female brains have gross differences in size and cell number.

Here, we examined only one genotype, a wild-type strain from the *Drosophila* Genetic Reference Panel (DGRP). Nonetheless, similar sexual dimorphism in adult brain size and numbers of cells in wandering 3^rd^ instar larval brains are observed in other strains indicating that sex-specific variation in overall brain size and cell number may be a widespread phenomenon [8,50]. Indeed, our findings further emphasize the need to analyze sex-specific differences in brain morphology at different scales to understand the functional consequences of differences in cell number. Recently, large-scale efforts have been taken to annotate and map the neuronal wiring in the whole brain of adult female [11–13] and male [51] Drosophila. Comparison of female and male brain connectomes revealed sexual dimorphism in neuronal connections; however, estimates of the total number of neurons in the brain were very similar in both the sexes [51]. This differs from what we observed here, and could be due to differences in methodology, genotype, cell types assayed, or inter-individual variability, among other possibilities. Future investigations would be needed to resolve these differences.

## 4. Materials and methods

### 4.1. Optimization of tagmentation reaction

We used inbred and wild-type *Drosophila melanogaster* line DRGP_373 from the *Drosophila* Genetic Reference Panel for this study [52,53]. Brains from 5−10 days old virgin adult *Drosophila* heads were dissected in cold ATAC-RSBT1 buffer (10 mM Tris-HCl pH 7.4, 10 mM NaCl, 3 mM MgCl_2_, 0.05% Tween-20 in H_2_O) and the optic lobes were removed with tweezers and a 28-gauge needle. After collecting 5 brains, the ATAC-RSBT1 was replaced by 500 μl of ATAC-RSBT2 (RSBT1 + 0.1% NP40, 0.1% Tween-20, and 0.01% Digitonin (G9441, Promega, Madison, USA) added on the day used). The brains were then transferred to a 2 ml Dounce homogenizer using a microscope ensure that all the brains were transferred. The brains were homogenized with the loose pestle (pestle A) for 25 strokes and then the tissue was incubated for 3 min before further homogenization with the tight pestle (pestle B) for 25 strokes. Quality of nuclei isolation was assessed at this stage by taking 20ul of the nuclei suspension and mixed with 2ul of Trypan blue stain (Catalog no: 15250−061, Gibco, Thermo Fisher Scientific, Waltham, USA). It was loaded on a disposable hemocytometer (Catalog No: DHC-B02-2, INCYTO, Chungnam-do, Republic of Korea,) to assess brain dissociation and nuclei isolation efficiency using automated Countess 3 cell counter (Thermo Fisher Scientific, Waltham, USA) (Figs 1 and S1). To proceed with tagmentation reaction, we used different amount of nuclei from 0.8 to 4.0 times of the central brain homogenate to a 1.5 ml microfuge tube and digitonin was diluted by adding 1 ml of ATAC-RSBT1. The required volume of male and female brain cell nuclei equivalent to varying proportions of the central brain (as detailed in Fig 2) were transferred to a new microfuge tube for the tagmentation reaction. The digitonin was further diluted by adding ATAC-RSBT1 to make the total volume 1.5 ml. The tube was inverted several times to mix the solution and then centrifuged at 1200g for 10 minutes to pellet the nuclei. The supernatant was carefully aspirated out so as not to lose the cell pellet on the wall of the tube. To avoid drying the pellet, it was immediately resuspended in 50 µl of transposition mixture (25 µl 2 × TD buffer, 16.5 µl 1 × PBS, 0.5 µl 1% digitonin, 0.5 µl 10% Tween-20, 5 µl sterile H_2_O and 2.5 µl transposase (20034210, Illumina, San Diego, USA)). The reaction was mixed with the pellet by pipetting. The tagmentation reaction was incubated at 37°C for 30 minutes in a thermomixer at 1000 RPM. Finally, tagmented DNA was purified by Zymo DNA Clean and Concentrator-5 Kit (Catalog D4014, Zymo research, Irvine, USA), and eluted in 22 µl of elution buffer. The tagmented DNA library was amplified using Illumina i5 and i7 index primers, and NEBNext High-Fidelity 2 × PCR Master Mix (Catalog M0541, NEB, Ipswich, USA) with following thermocycler conditions: one cycle of 72°C for 5 min, 98°C for 30 sec, followed by 13 cycles of [98°C for 10 sec, 63°C for 30 sec, 72°C for 1 min], and then hold at 4°C. The pattern of the amplified library was tested using a Bioanalyzer 2100 (Agilent, Santa Clara, USA).

### 4.2. Nuclei isolation and sample preparation

Five brains of each sex were isolated in cold 1 × PBS in a dissection dish. PBS was carefully replaced by 100 μl of nuclei isolation buffer (NIB: 10 mM Tris–HCl pH 7.5, 10 mM NaCl, 3 mM MgCl_2_, 3 mM CaCl_2,_ 0.1% Tween-20, 0.1% NP40 substitute, 0.1% TritinX-100 and 0.01% digitonin). Brains were transferred to a 1.5 ml microfuge tube and gently homogenized with a 1.5 ml hand pestle for 10–15 strokes. 15 μl of collagenase Type IV (1000 U/μl stock, Catalog# 17104019 Gibco, Waltham, USA) was added and incubated at 37°C for 1 hour at 1000 rpm shaking on a thermomixer. After collagenase incubation, 360 μl of NIB with 2% BSA (NIB2) and 25 μl of counting beads were added to raise the total volume to 500 μl to dilute the nuclei to 100 μl/brain. After this step, the pipette tip was always rinsed with NIB2 several times by pipetting in and out before using it for the nuclei handling. Finally, 1 μl of fluorescent DNA binding dye (either Propidium Iodide (PI) or 4′,6-diamidino-2-phenylindole (DAPI) Catalog P3566 and Catalog-62248 respectively from Thermo Fisher Scientific, Waltham, USA) was added to the samples and incubated at room temperature for at least 30 minutes before flow cytometer counting. A 96 well polypropylene plate was prepared for counting nuclei from multiple samples. The desired volume of the isolated nuclei was added to the sample along with enough NIB2 to make a final volume of 200 μl. To avoid any carry over of nuclei between the samples, each sample well was separated by four wells having 150 μl of 10% bleach, 4% Contrad detergent, water and NIB2 to wash the flow cytometer probe and connected tubing.

### 4.3. Fluorescence-activated cell sorting (FACS) setup and nuclei counting

A high throughput 96 well sampler (Becton Dickinson Biosciences, Franklin Lakes, USA) attached to the cytometer (Becton Dickinson Biosciences, Franklin Lakes, USA) was programmed to mix the samples in a 96 well plate before counting. Only 150 μl of the total 200 μl was used for counting to ensure that the same volume was recorded for each sample. HTS probe was washed with 10% bleach (Clorox, Oakland, USA), 4% Contrad-70 detergent (Deconlabs, Bryn Mawr, USA), water and NIB2, respectively, to ensure no sample-to-sample carryover. The BD LSR-2 Fortessa analytical Cytometer equipped with 355, 407, 488, 532 and 640 nm laser lines using BDFACSDiva software (Becton Dickinson, Franklin Lakes, USA) was used for DAPI- and PI-based counting of nuclei. The PI signal was recorded using 532 nm excitation and emission was collected at the 610−40nm bandpass filter, while DAPI signal was recorded using 407 excitation while emission was recorded at the 450−50nm bandpass filter. Data recording was thresholded for DAPI or PI fluorescence intensity at 500 to minimize the cell debris and background counting. We used Flow-Jo version 10 software (Becton Dickinson Biosciences, Franklin Lakes, USA) with same gating parameters to count number of nuclei and singlets across the experiments.

**4.4. Brain weight and size analyses:** Whole fly images were taken of anesthetized flies directly on the CO_2_ pad, while heads were decapitated and both sexes were front imaged at same magnification. To weigh the heads, we first weighed an empty microfuge tube, then added 30 decapitated heads and weighed it again. This was repeated three times to get an average weight of 30 heads. To estimate the size (area) of the brain, each brain was dissected gently to make sure it did not pull and lose its shape. Then it was mounted on a slide using a coverslip with a spacer to avoid changes in the shape of brain due to weight of the coverslip. The images of the mounted brains (13 of females and 12 of males) were taken images at 20X magnification and area was measured using Leica stereo-camera Enersight software. The statistical analyses were done using SAS software.

## Supporting information

S1 Fig Brain cell dissociation analyses. (PDF)

S2 Fig Overlay image of tagmentation. (PDF)

S3 Fig Analysis of nuclei suspension for singlets. (PDF)

S1 Table Data values for all traits in the experiment. (XLSX)

S2 Table ANOVA table for all traits. (XLSX)

S1 Raw Images Raw images used to generate Fig 2. (PDF)
