# Supplementary material for: Sexual dimorphism in the cell number of the adult Drosophila brain
Source: PLoS One. 2026 Feb 18;21(2):e0342456. doi: 10.1371/journal.pone.0342456 (PMC12915905; doi:10.1371/journal.pone.0342456)

Supplementary figure 1: Brain cell dissociation analyses:

Adult Drosophila brains were dissociated using the protocol described in the manuscript. Brain dissociation into nuclei was analyzed by adding 2 μL of Trypan Blue to 18 μL of the nuclei suspension, followed by loading the mixture onto a disposable hemocytometer. The nuclei suspension was analyzed using the Countess 3 automated cell counter. Data from two female and two male samples are presented below:

A) Female sample 1

Total		2.42×10^6/mL
Live	7%	1.64×10^5/mL
Dead	93%	2.26×10^6/mL

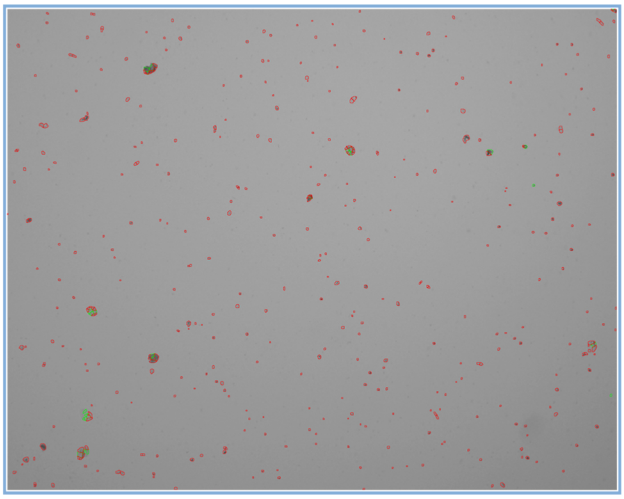

B) Female sample 2

Total		1.91×10^6/mL
Live	8%	1.47×10^5/mL
Dead	92%	1.76×10^6/mL

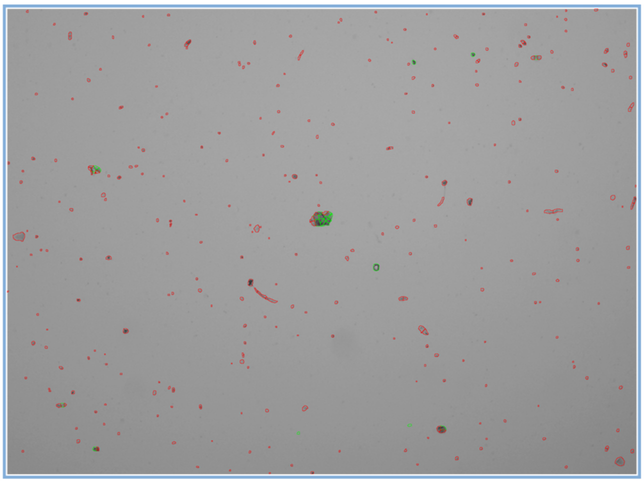

C) Male sample 1

Total		2.08×10^6/mL
Live	5%	9.38×10^4/mL
Dead	95%	1.99×10^6/mL

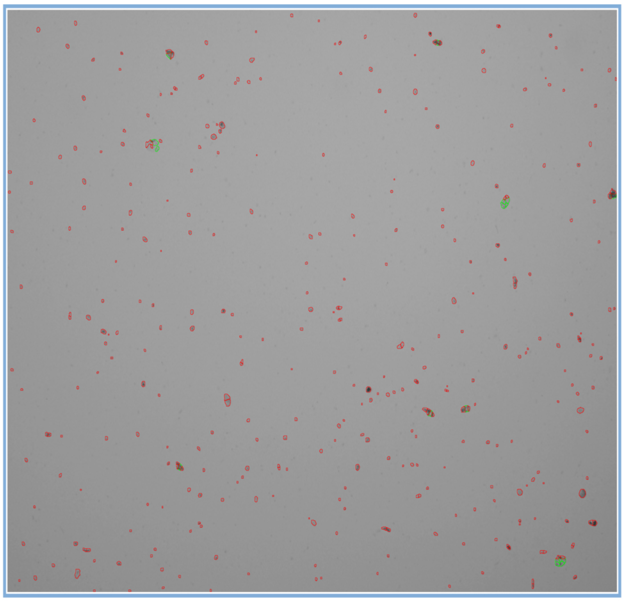

D) Male sample 2

Total		2.06×10^6/mL
Live	3%	5.28×10^4/mL
Dead	97%	2.01×10^6/mL

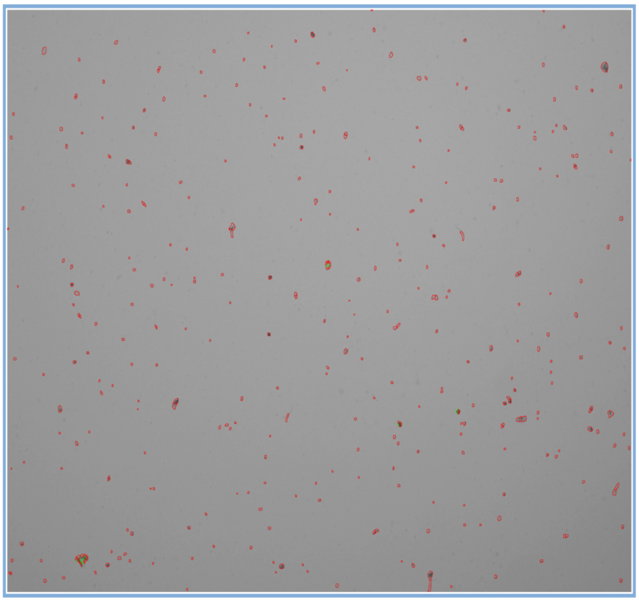

Supplement: S1 Fig — (PDF) [file pone.0342456.s001.pdf]
