# Supplementary material for: Sexual dimorphism in the cell number of the adult Drosophila brain
Source: PLoS One. 2026 Feb 18;21(2):e0342456. doi: 10.1371/journal.pone.0342456 (PMC12915905; doi:10.1371/journal.pone.0342456)

Supplementary Figure 2: Overlay of Tagmented DNA: Bioanalyzer traces of tagmented DNA from 1.75X and 2X male brain, and 1.35X and 1.5X female brain nuclei are overlaid to show the distribution of nucleosome-free regions, mono-, di-, and tri-nucleosome sizes. The Y-axis represents fluorescence intensity, while the X-axis represents DNA size in base pairs.

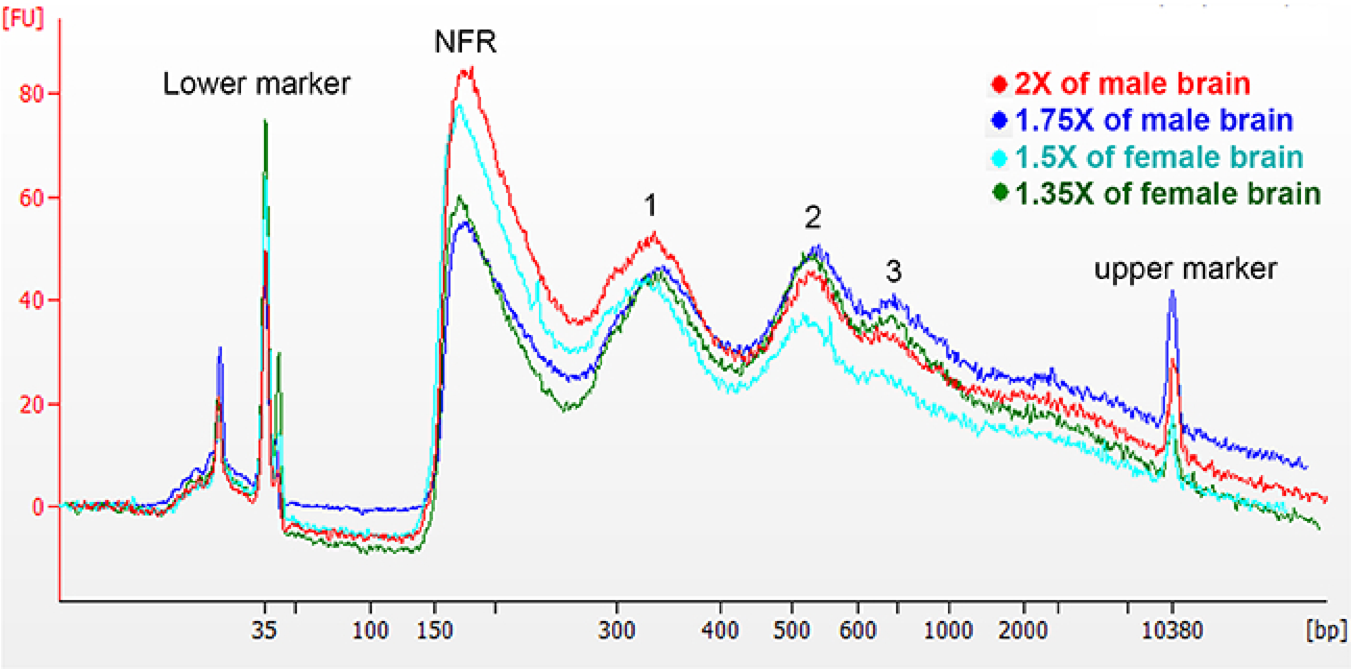

Supplement: S2 Fig — (PDF) [file pone.0342456.s002.pdf]
