# Supplementary material for: Sexual dimorphism in the cell number of the adult Drosophila brain
Source: PLoS One. 2026 Feb 18;21(2):e0342456. doi: 10.1371/journal.pone.0342456 (PMC12915905; doi:10.1371/journal.pone.0342456)
Supplement: S3 Fig — (PDF) [file pone.0342456.s003.pdf]

Supplementary Figure 3A–D: Analysis of nuclei suspension for singlets

Nuclei isolation and flow cytometry analysis were performed using the protocol described in the main manuscript. Nuclei were analyzed for the presence of singlets in the nuclei counting data using FlowJo version 10 software.

A) Female brain nuclei stained with Propidium Iodide (PI):
i) A bivariate plot of DAPI vs. PI shows that the majority of nuclei (>99%) are stained with PI, with a small background signal (~0.16%) observed in the DAPI channel.
ii) A bivariate plot of area vs. width indicates that over 99% of PI-positive nuclei are singlets.
iii) The summary table presents the percentage and number of nuclei stained with each dye, along with the proportion of singlets.

i)

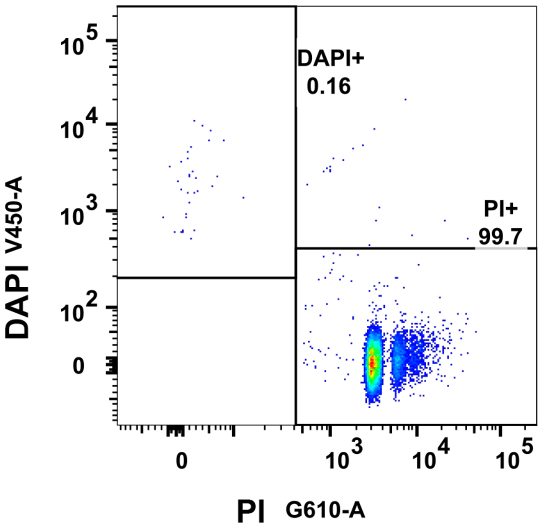

ii)

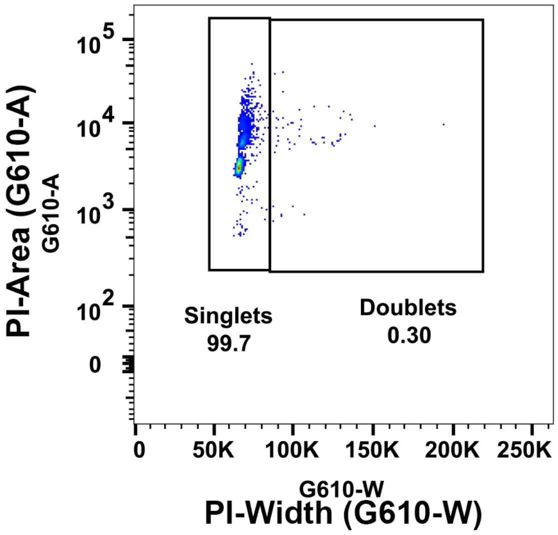

iii)

Subset Name	Freq. of Parent	Count
Events	94.0	19573
DAPI+	0.16	32.0
Doublets	34.4	11.0
Singlets	65.6	21.0
PI+	99.7	19523
Doublets	0.30	58.0
Singlets	99.7	19465

B) Male brain nuclei stained with Propidium Iodide (PI):

i) A bivariate plot of DAPI vs. PI shows that the majority of nuclei (>99%) are stained with PI, with a small background signal (~0.23%) observed in the DAPI channel.

ii) A bivariate plot of area vs. width indicates that over 99% of PI-positive nuclei are singlets.

iii) The summary table presents the percentage and number of nuclei stained with each dye, along with the proportion of singlets.

i)

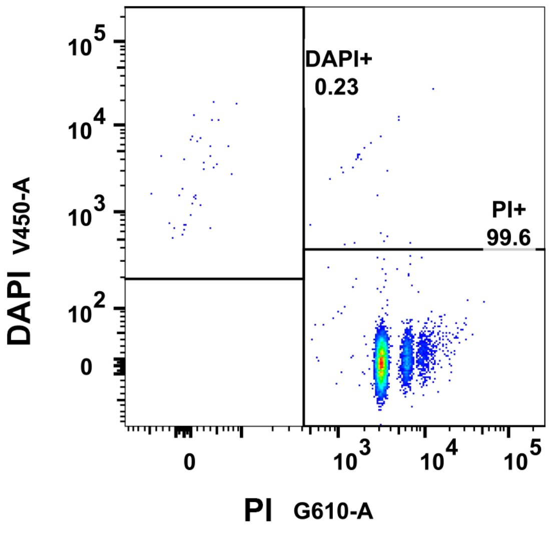

ii)

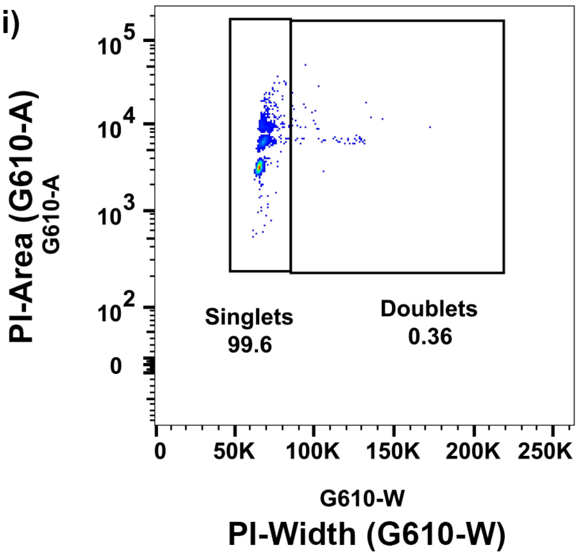

iii)

Subset Name	Freq. of Parent	Count
Events	93.7	15837
DAPI+	0.23	37.0
Doublets	32.4	12.0
Singlets	67.6	25.0
PI+	99.6	15775
Doublets	0.36	57.0
Singlets	99.6	15718

C) Female brain nuclei stained with 4',6-diamidino-2-phenylindole (DAPI):
i) A bivariate plot of DAPI vs. PI shows that all the nuclei (100%) are stained with DAPI, with no background signal observed in the PI channel.
ii) A bivariate plot of area vs. width indicates that over 98% of DAPI-positive nuclei are singlets.
iii) The summary table presents the percentage and number of nuclei stained with each dye, along with the proportion of singlets.

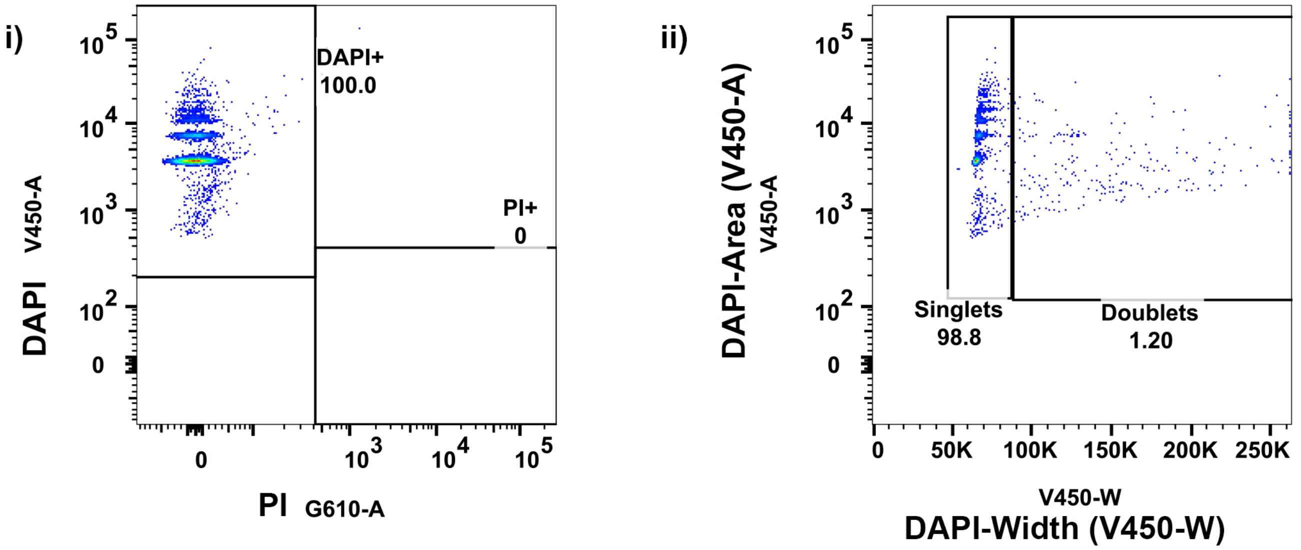

iii)

Subset Name	Freq. of Parent	Count
Events	93.1	19608
DAPI+	100.0	19600
Doublets	1.20	236
Singlets	98.8	19358
PI+	0	0
Doublets	0	0
Singlets	0	0

D) Male brain nuclei stained with 4',6-diamidino-2-phenylindole (DAPI):
i) A bivariate plot of DAPI vs. PI shows that most of the nuclei (99.9%) are stained with DAPI, with no background signal observed in the PI channel.
ii) A bivariate plot of area vs. width indicates that over 99% of DAPI-positive nuclei are singlets.
iii) The summary table presents the percentage and number of nuclei stained with each dye, along with the proportion of singlets.

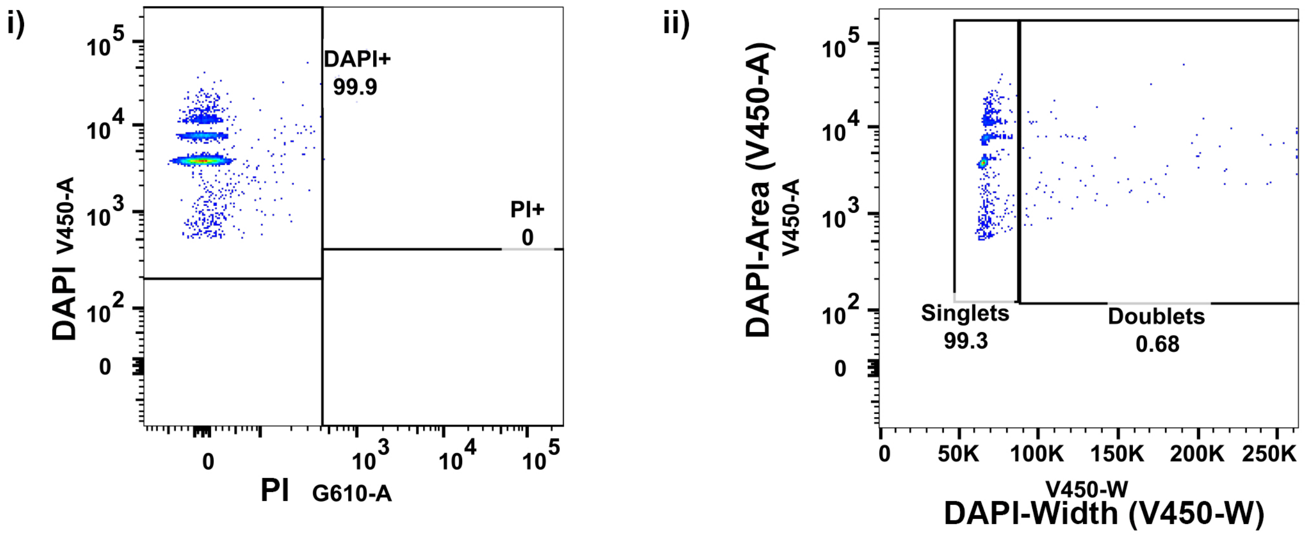

iii)

Subset Name	Freq. of Parent	Count
Events	93.3	14596
DAPI+	99.9	14586
Doublets	0.68	99.0
Singlets	99.3	14485
PI+	0	0
Doublets	0	0
Singlets	0	0
