# Supplementary material for: Sexual dimorphism in the cell number of the adult Drosophila brain
Source: PLoS One. 2026 Feb 18;21(2):e0342456. doi: 10.1371/journal.pone.0342456 (PMC12915905; doi:10.1371/journal.pone.0342456)
Supplement: S1 Raw Images — (PDF) [file pone.0342456.s006.pdf]

Assay Class:	High Sensitivity DNA Assay	Created:	3/8/2022 1:08:50 PM
Data Path:	D:\...gh Sensitivity DNA Assay_DEDAE01941_2022-03-08_13-08-51.xad	Modified:	9/11/2025 2:56:08 PM

Electrophoresis File Run Summary

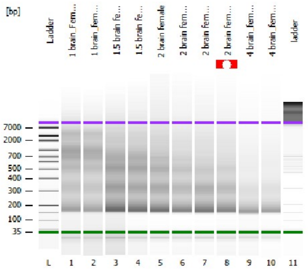

Instrument Information:

Instrument Name:	DEDAE01941	Firmware:	C.01.069
Serial#:	DEDAE01941	Type:	G2939B

Assay Information:

Assay Origin Path:	C:\Program Files (x86)\Agilent\2100 bioanalyzer\2100 expert\assays\dsDNA\High Sensitivity DNA.xsy
Assay Class:	High Sensitivity DNA Assay
Version:	1.03
Assay Comments:	Copyright © 2003-2010 Agilent Technologies

Chip Information:

Chip Lot #:

Reagent Kit Lot #:

Chip Comments:

1 brain_Female

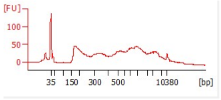

1 brain_female

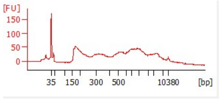

1.5 brain female

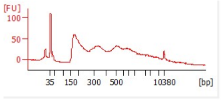

1.5 brain female

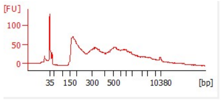

2 brain female

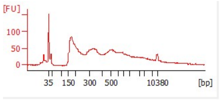

2 brain female 2

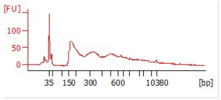

2 brain female replicate

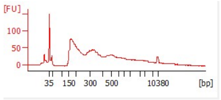

2 brain female replicate 2

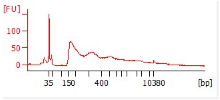

4 brain_female

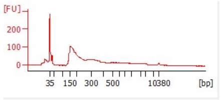

4 brain_female replicate

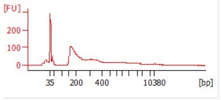

ladder

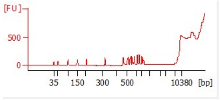

 © Copyright 2003 - 2018 Agilent, Inc.

Assay Class: High Sensitivity DNA Assay
Data Path: D:\...gh Sensitivity DNA Assay_DEDAE01941_2022-03-08_13-08-51.xad

Created: 3/8/2022 1:08:50 PM
Modified: 9/11/2025 2:56:08 PM

Electrophoresis File Run Summary (Chip Summary)

Sample Name	Sample Comment	Rest. Digest	Status	Observation	Result Label	Result Color
1 brain_Female		☐	✓			
1 brain_female		☐	✓			
1.5 brain female		☐	✓			
1.5 brain female		☐	✓			
2 brain female		☐	✓			
2 brain female 2		☐	✓			
2 brain female replicate		☐	✓			
2 brain female replicate		☐	✓			
4 brain_female		☐	✓			
4 brain_female replicate		☐	✓			
ladder		☐	✓			
Ladder		☐	✓			

Chip Lot #

Reagent Kit Lot #

Chip Comments :

© Copyright 2003 - 2018 Agilent, Inc.

Assay Class: High Sensitivity DNA Assay
Data Path: D:\...gh Sensitivity DNA Assay_DEDAE01941_2022-03-08_13-08-51.xad
Created: 3/8/2022 1:08:50 PM
Modified: 9/11/2025 2:56:08 PM

Electrophoresis Assay Details

General Analysis Settings

Number of Available Sample and Ladder Wells (Max.) : 12
Minimum Visible Range [s] : 32
Maximum Visible Range [s] : 138
Start Analysis Time Range [s] : 33
End Analysis Time Range [s] : 137.5
Ladder Concentration [pg/µl] : 1950
Uses Standard Area for Ladder Fragments
Lower Marker Concentration [pg/µl] : 125
Upper Marker Concentration [pg/µl] : 75
Used Upper Marker for Quantitation
Standard Curve Fit is Point to Point
Show Data Aligned to Lower and Upper Marker

Integrator Settings

Integration Start Time [s] : 33.05
Integration End Time [s] : 137
Slope Threshold : 0.8
Height Threshold [FU] : 5
Area Threshold : 0.1
Width Threshold [s] : 0.6
Baseline Plateau [s] : 0.5

Filter Settings

Filter Width [s] : 0.5
Polynomial Order : 4

Ladder

Ladder Peak	Size	Area
1	35	160
2	50	210
3	100	208
4	150	221
5	200	242
6	300	270
7	400	305
8	500	306
9	600	336
10	700	321
11	1000	366
12	2000	413
13	3000	411
14	7000	400
15	10380	214

© Copyright 2003 - 2018 Agilent, Inc.

Assay Class:	High Sensitivity DNA Assay	Created:	3/8/2022 1:08:50 PM
Data Path:	D:\...gh Sensitivity DNA Assay_DEDAE01941_2022-03-08_13-08-51.xad	Modified:	9/11/2025 2:56:08 PM

Electropherogram Summary

Ladder

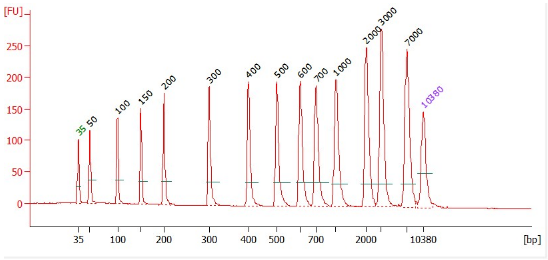

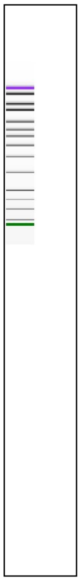

Overall Results for Ladder

Noise: 0.3

Peak table for Ladder

Peak		Size [bp]	Conc. [pg/µl]	Molarity [pmol/l]	Observations
2		50	150.00	4,545.5	Ladder Peak
3		100	150.00	2,272.7	Ladder Peak
4		150	150.00	1,515.2	Ladder Peak
5		200	150.00	1,136.4	Ladder Peak
6		300	150.00	757.6	Ladder Peak
7		400	150.00	568.2	Ladder Peak
8		500	150.00	454.5	Ladder Peak
9		600	150.00	378.8	Ladder Peak
10		700	150.00	324.7	Ladder Peak
11		1,000	150.00	227.3	Ladder Peak
12		2,000	150.00	113.6	Ladder Peak
13		3,000	150.00	75.8	Ladder Peak
14		7,000	150.00	32.5	Ladder Peak

 © Copyright 2003 - 2018 Agilent, Inc.

Assay Class: High Sensitivity DNA Assay
Data Path: D:\...gh Sensitivity DNA Assay_DEDAE01941_2022-03-08_13-08-51.xad

Created: 3/8/2022 1:08:50 PM
Modified: 9/11/2025 2:56:08 PM

Electropherogram Summary Continued ...

1 brain_Female

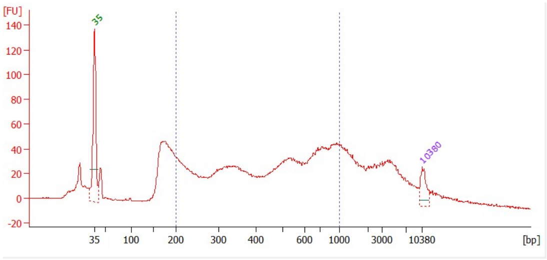

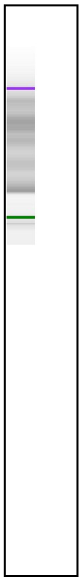

Overall Results for sample 1 : __1 brain_Female__

Number of peaks found: 0

Corr. Area 1: 1,285.9

Noise: 0.4

Region table for sample 1 : __1 brain_Female__

From [bp]	To [bp]	Corr. Area	% of Total	Average Size [bp]	Size distribution in CV [%]	Conc. [pg/µl]	Molarity [pmol/l]	Co lor
200	1,000	1,285.9	56	499	43.1	3,430.48	13,811.3	

© Copyright 2003 - 2018 Agilent, Inc.

Assay Class: High Sensitivity DNA Assay
Data Path: D:\...gh Sensitivity DNA Assay_DEDAE01941_2022-03-08_13-08-51.xad

Created: 3/8/2022 1:08:50 PM
Modified: 9/11/2025 2:56:08 PM

Electropherogram Summary Continued ...

1 brain_female

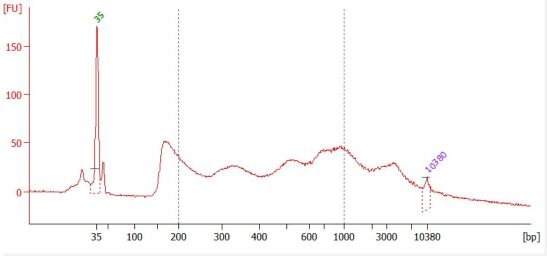

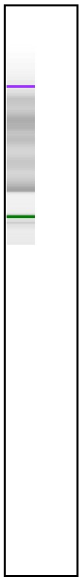

Overall Results for sample 2 : **1 brain_female**

Number of peaks found: 0

Corr. Area 1: 1,416.2

Noise: 0.7

Region table for sample 2 : **1 brain_female**

From [bp]	To [bp]	Corr. Area	% of Total	Average Size [bp]	Size distribution in CV [%]	Conc. [pg/µl]	Molarity [pmol/l]	Color
200	1,000	1,416.2	56	502	42.8	3,771.05	15,082.0	

© Copyright 2003 - 2018 Agilent, Inc.

Assay Class: High Sensitivity DNA Assay
Data Path: D:\...gh Sensitivity DNA Assay_DEDAE01941_2022-03-08_13-08-51.xad

Created: 3/8/2022 1:08:50 PM
Modified: 9/11/2025 2:56:08 PM

Electropherogram Summary Continued ...

1.5 brain female

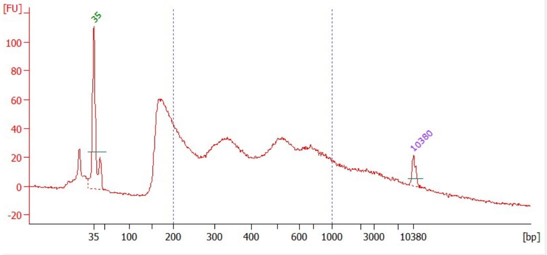

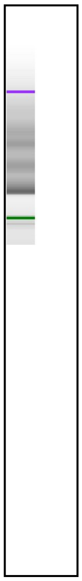

Overall Results for sample 3 : 1.5 brain female

Number of peaks found: 0

Corr. Area 1: 1,393.6

Noise: 0.5

Region table for sample 3 : 1.5 brain female

From [bp]	To [bp]	Corr. Area	% of Total	Average Size [bp]	Size distribution in CV [%]	Conc. [pg/µl]	Molarity [pmol/l]	Co lor
200	1,000	1,393.6	63	449	42.6	8,845.93	38,165.2	

 © Copyright 2003 - 2018 Agilent, Inc.

Assay Class: High Sensitivity DNA Assay
Data Path: D:\...gh Sensitivity DNA Assay_DEDAE01941_2022-03-08_13-08-51.xad

Created: 3/8/2022 1:08:50 PM
Modified: 9/11/2025 2:56:08 PM

Electropherogram Summary Continued ...

1.5 brain female

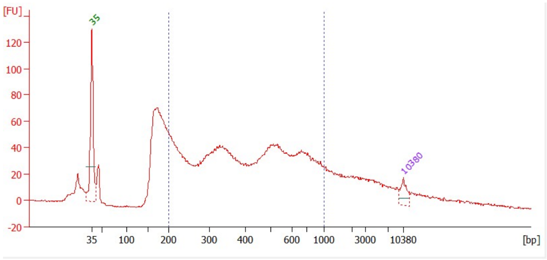

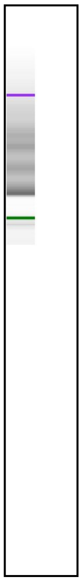

Overall Results for sample 4 : 1.5 brain female

Number of peaks found: 0

Corr. Area 1: 1,557.7

Noise: 0.6

Region table for sample 4 : 1.5 brain female

From [bp]	To [bp]	Corr. Area	% of Total	Average Size [bp]	Size distribution in CV [%]	Conc. [pg/µl]	Molarity [pmol/l]	Co lor
200	1,000	1,557.7	62	451	42.4	5,989.55	25,711.1	

© Copyright 2003 - 2018 Agilent, Inc.

Assay Class: High Sensitivity DNA Assay
Data Path: D:\...gh Sensitivity DNA Assay_DEDAE01941_2022-03-08_13-08-51.xad
Created: 3/8/2022 1:08:50 PM
Modified: 9/11/2025 2:56:08 PM

Electropherogram Summary Continued ...

2 brain female

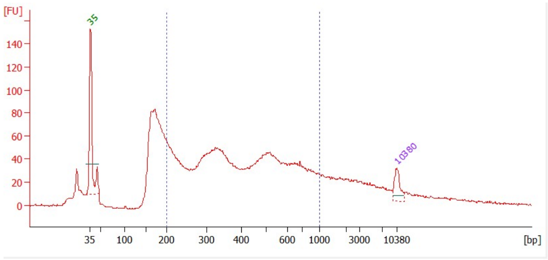

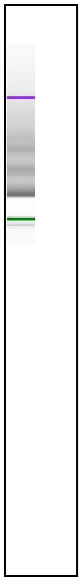

Overall Results for sample 5 : 2 brain female

Number of peaks found: 0
Corr. Area 1: 1,617.4
Noise: 0.7

Region table for sample 5 : 2 brain female

From [bp]	To [bp]	Corr. Area	% of Total	Average Size [bp]	Size distribution in CV [%]	Conc. [pg/µl]	Molarity [pmol/l]	Co lor
200	1,000	1,617.4	61	437	42.4	4,737.82	20,707.6	

© Copyright 2003 - 2018 Agilent, Inc.

Assay Class: High Sensitivity DNA Assay
Data Path: D:\...gh Sensitivity DNA Assay_DEDAE01941_2022-03-08_13-08-51.xad

Created: 3/8/2022 1:08:50 PM
Modified: 9/11/2025 2:56:08 PM

Electropherogram Summary Continued ...

2 brain female 2

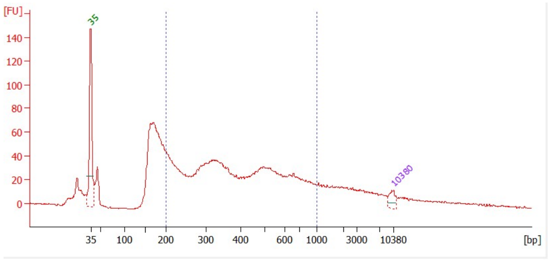

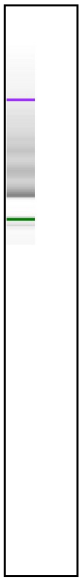

Overall Results for sample 6 : 2 brain female 2

Number of peaks found: 0
Corr. Area 1: 1,199.5
Noise: 0.5

Region table for sample 6 : 2 brain female 2

From [bp]	To [bp]	Corr. Area	% of Total	Average Size [bp]	Size distribution in CV [%]	Conc. [pg/µl]	Molarity [pmol/l]	Co lor
200	1,000	1,199.5	59	428	42.4	6,360.93	28,241.1	

 © Copyright 2003 - 2018 Agilent, Inc.

Figure 2A_Female_2x_Brain

Assay Class: High Sensitivity DNA Assay
Data Path: D:\...gh Sensitivity DNA Assay_DEDAE01941_2022-03-08_13-08-51.xad

Created: 3/8/2022 1:08:50 PM
Modified: 9/11/2025 2:56:08 PM

Electropherogram Summary Continued ...

2 brain female replicate

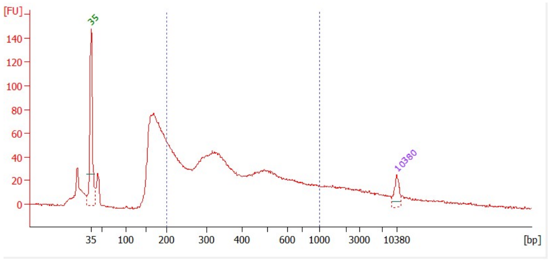

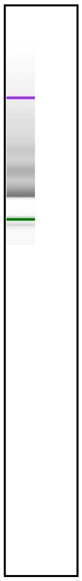

Overall Results for sample 7 : <u>2 brain female replicate</u>

Number of peaks found: 0 Corr. Area 1: 1,335.8

Noise: 0.6

Region table for sample 7 : <u>2 brain female replicate</u>

From [bp]	To [bp]	Corr. Area	% of Total	Average Size [bp]	Size distribution in CV [%]	Conc. [pg/µl]	Molarity [pmol/l]	Color
200	1,000	1,335.8	59	407	43.2	4,924.20	22,745.4	

 © Copyright 2003 - 2018 Agilent, Inc.

Assay Class: High Sensitivity DNA Assay
Data Path: D:\...gh Sensitivity DNA Assay_DEDAE01941_2022-03-08_13-08-51.xad
Created: 3/8/2022 1:08:50 PM
Modified: 9/11/2025 2:56:08 PM

Electropherogram Summary Continued ...

2 brain female replicate 2

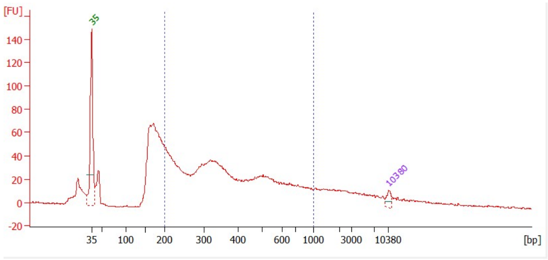

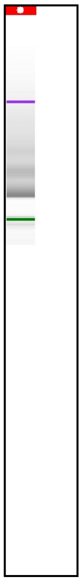

Overall Results for sample 8 : 2 brain female replicate 2

Number of peaks found: 0
Corr. Area 1: 1,110.0
Noise: 0.5

Region table for sample 8 : 2 brain female replicate 2

From [bp]	To [bp]	Corr. Area	% of Total	Average Size [bp]	Size distribution in CV [%]	Conc. [pg/µl]	Molarity [pmol/l]	Color
200	1,000	1,110.0	58	405	43.3	7,810.01	36,233.5	

© Copyright 2003 - 2018 Agilent, Inc.

Assay Class: High Sensitivity DNA Assay
Data Path: D:\...gh Sensitivity DNA Assay_DEDAE01941_2022-03-08_13-08-51.xad
Created: 3/8/2022 1:08:50 PM
Modified: 9/11/2025 2:56:08 PM

Electropherogram Summary Continued ...

4 brain_female

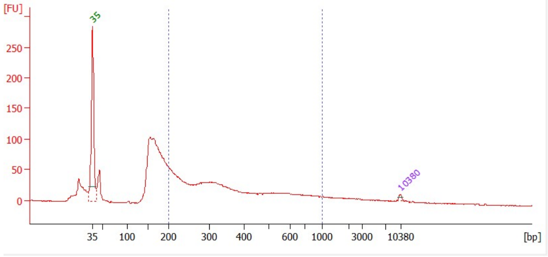

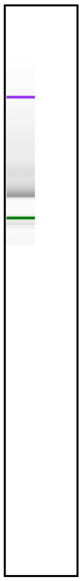

Overall Results for sample 9 : 4 brain_female

Number of peaks found: 0
Corr. Area 1: 1,006.0
Noise: 0.6

Region table for sample 9 : 4 brain_female

From [bp]	To [bp]	Corr. Area	% of Total	Average Size [bp]	Size distribution in CV [%]	Conc. [pg/µl]	Molarity [pmol/l]	Color
200	1,000	1,006.0	48	384	45.5	19,281.11	94,655.6	

 © Copyright 2003 - 2018 Agilent, Inc.

Assay Class: High Sensitivity DNA Assay Created: 3/8/2022 1:08:50 PM
Data Path: D:\...gh Sensitivity DNA Assay_DEDAE01941_2022-03-08_13-08-51.xad Modified: 9/11/2025 2:56:08 PM

Electropherogram Summary Continued ...

4 brain_female replicate

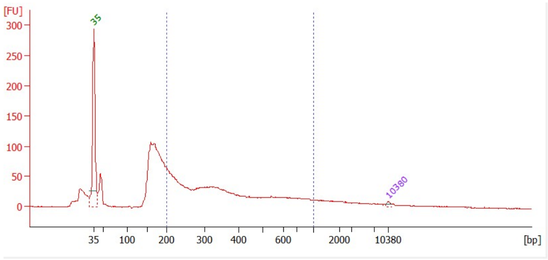

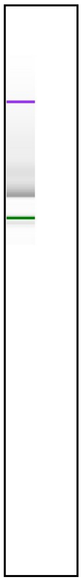

Overall Results for sample 10 : **4 brain female replicate**

Number of peaks found: 0 Corr. Area 1: 1,085.8

Noise: 0.5

Region table for sample 10 : **4 brain female replicate**

From [bp]	To [bp]	Corr. Area	% of Total	Average Size [bp]	Size distribution in CV [%]	Conc. [pg/µl]	Molarity [pmol/l]	Co lor
200	1,000	1,085.8	50	386	45.8	15,074.95	73,886.8	

 © Copyright 2003 - 2018 Agilent, Inc.

Assay Class: High Sensitivity DNA Assay
Data Path: D:\...gh Sensitivity DNA Assay_DEDAE01941_2022-03-08_13-08-51.xad

Created: 3/8/2022 1:08:50 PM
Modified: 9/11/2025 2:56:08 PM

Gel Image

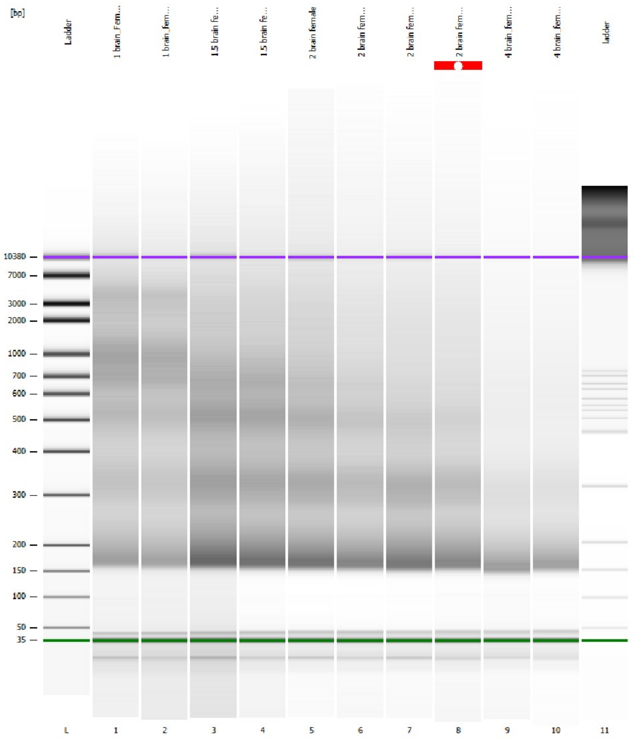

 © Copyright 2003 - 2018 Agilent, Inc.

Assay Class: High Sensitivity DNA Assay
Data Path: D:\...gh Sensitivity DNA Assay_DEDAE01941_2022-03-08_13-08-51.xad
Created: 3/8/2022 1:08:50 PM
Modified: 9/11/2025 2:56:08 PM

Run Logbook

Description	Number	Source	Category	Sub Category	Time	Time Zone	User	Host
Run ended on port 3 (Number of wells acquired: 12)		Instrument	Run		3/8/2022 1:50:10 PM	(GMT --05:00) Eastern Standard Time	harbison_lab	SBC-XL02213361
Run started on port 3 (File: C:\Program Files (x86)\Agilent\2100 bioanalyzer\2100 expert\Data\2022-03-08\2100 expert_High Sensitivity DNA Assay_DEDAE01941_2022-03-08_13-08-51.xad)		Instrument	Run		3/8/2022 1:08:56 PM	(GMT --05:00) Eastern Standard Time	harbison_lab	SBC-XL02213361
Product Number : G2939B		Instrument	Run		3/8/2022 1:08:56 PM	(GMT --05:00) Eastern Standard Time	harbison_lab	SBC-XL02213361
Name :		Instrument	Run		3/8/2022 1:08:56 PM	(GMT --05:00) Eastern Standard Time	harbison_lab	SBC-XL02213361
Vendor : Agilent Technologies		Instrument	Run		3/8/2022 1:08:56 PM	(GMT --05:00) Eastern Standard Time	harbison_lab	SBC-XL02213361
Serial# : DEDAE01941		Instrument	Run		3/8/2022 1:08:56 PM	(GMT --05:00) Eastern Standard Time	harbison_lab	SBC-XL02213361
Firmware : C.01.069		Instrument	Run		3/8/2022 1:08:56 PM	(GMT --05:00) Eastern Standard Time	harbison_lab	SBC-XL02213361
Cartridge : Electrode		Instrument	Run		3/8/2022 1:08:56 PM	(GMT --05:00) Eastern Standard Time	harbison_lab	SBC-XL02213361

© Copyright 2003 - 2018 Agilent, Inc.

Assay Class: High Sensitivity DNA Assay
Data Path: D:\...gh Sensitivity DNA Assay_DEDAE01941_2022-03-30_15-45-09.xad
Created: 3/30/2022 3:45:08 PM
Modified: 9/11/2025 1:45:09 PM

Electrophoresis File Run Summary

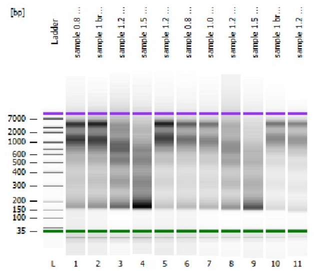

Instrument Information:

Instrument Name: DEDAE01941 Firmware: C.01.069

Serial#: DEDAE01941 Type: G2939B

Assay Information:

Assay Origin Path: C:\Program Files (x86)\Agilent\2100 bioanalyzer\2100 expert\assays\dsDNA\High Sensitivity DNA.xsy

Assay Class: High Sensitivity DNA Assay

Version: 1.03

Assay Comments: Copyright © 2003-2010 Agilent Technologies

Chip Information:

Chip Lot #:

Reagent Kit Lot #:

Chip Comments:

sample 0.8 brain female

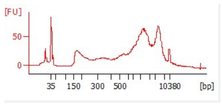

sample 1 brain female

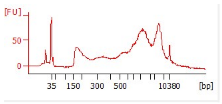

sample 1.2 brain Female

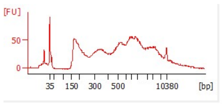

sample 1.5 brain female

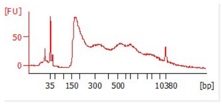

sample 1.2 brain male

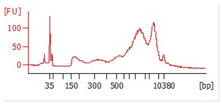

sample 0.8 brain female 2

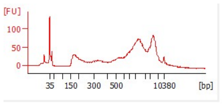

sample 1.0 brain female 2

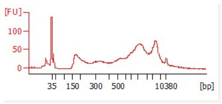

sample 1.2 brain female 2

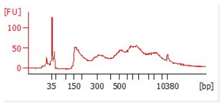

sample 1.5 brain female 2

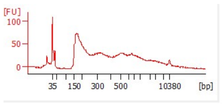

sample 1 brain male

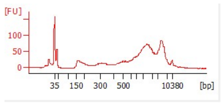

sample 1.2 brain male

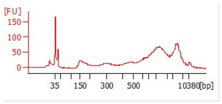

Assay Class: High Sensitivity DNA Assay
Data Path: D:\...gh Sensitivity DNA Assay_DEDAE01941_2022-03-30_15-45-09.xad
Created: 3/30/2022 3:45:08 PM
Modified: 9/11/2025 1:45:09 PM

Electrophoresis File Run Summary (Chip Summary)

Sample Name	Sample Comment	Rest. Digest	Status	Observation	Result Label	Result Color
sample 0.8 brain female		☐	✓			
sample 1 brain female		☐	✓			
sample 1.2 brain Female		☐	✓			
sample 1.5 brain female		☐	✓			
sample 1.2 brain male		☐	✓			
sample 0.8 brain female		☐	✓			
sample 1.0 brain female		☐	✓			
sample 1.2 brain female		☐	✓			
sample 1.5 brain female		☐	✓			
sample 1 brain male		☐	✓			
sample 1.2 brain male		☐	✓			
Ladder		☐	✓			

Chip Lot # **Reagent Kit Lot #**

Chip Comments :

© Copyright 2003 - 2018 Agilent, Inc.

Assay Class: High Sensitivity DNA Assay
Data Path: D:\...gh Sensitivity DNA Assay_DEDAE01941_2022-03-30_15-45-09.xad
Created: 3/30/2022 3:45:08 PM
Modified: 9/11/2025 1:45:09 PM

Electrophoresis Assay Details

General Analysis Settings

Number of Available Sample and Ladder Wells (Max.) : 12
Minimum Visible Range [s] : 32
Maximum Visible Range [s] : 138
Start Analysis Time Range [s] : 33
End Analysis Time Range [s] : 137.5
Ladder Concentration [pg/µl] : 1950
Uses Standard Area for Ladder Fragments
Lower Marker Concentration [pg/µl] : 125
Upper Marker Concentration [pg/µl] : 75
Used Upper Marker for Quantitation
Standard Curve Fit is Point to Point
Show Data Aligned to Lower and Upper Marker

Integrator Settings

Integration Start Time [s] : 33.05
Integration End Time [s] : 137
Slope Threshold : 0.8
Height Threshold [FU] : 5
Area Threshold : 0.1
Width Threshold [s] : 0.6
Baseline Plateau [s] : 0.5

Filter Settings

Filter Width [s] : 0.5
Polynomial Order : 4

Ladder

Ladder Peak	Size	Area
1	35	160
2	50	210
3	100	208
4	150	221
5	200	242
6	300	270
7	400	305
8	500	306
9	600	336
10	700	321
11	1000	366
12	2000	413
13	3000	411
14	7000	400
15	10380	214

 © Copyright 2003 - 2018 Agilent, Inc.

Assay Class:	High Sensitivity DNA Assay	Created:	3/30/2022 3:45:08 PM
Data Path:	D:\...gh Sensitivity DNA Assay_DEDAE01941_2022-03-30_15-45-09.xad	Modified:	9/11/2025 1:45:09 PM

Electropherogram Summary

Ladder

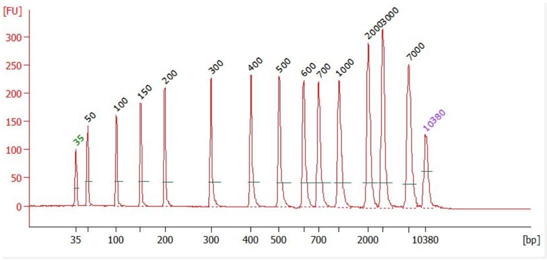

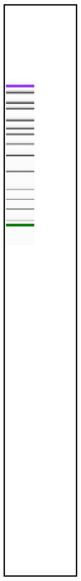

Overall Results for Ladder

Noise: 0.3

Peak table for Ladder

Peak	Size [bp]	Conc. [pg/µl]	Molarity [pmol/l]	Observations
2	50	150.00	4,545.5	Ladder Peak
3	100	150.00	2,272.7	Ladder Peak
4	150	150.00	1,515.2	Ladder Peak
5	200	150.00	1,136.4	Ladder Peak
6	300	150.00	757.6	Ladder Peak
7	400	150.00	568.2	Ladder Peak
8	500	150.00	454.5	Ladder Peak
9	600	150.00	378.8	Ladder Peak
10	700	150.00	324.7	Ladder Peak
11	1,000	150.00	227.3	Ladder Peak
12	2,000	150.00	113.6	Ladder Peak
13	3,000	150.00	75.8	Ladder Peak
14	7,000	150.00	32.5	Ladder Peak

 © Copyright 2003 - 2018 Agilent, Inc.

Assay Class: High Sensitivity DNA Assay
Data Path: D:\...gh Sensitivity DNA Assay_DEDAE01941_2022-03-30_15-45-09.xad

Created: 3/30/2022 3:45:08 PM
Modified: 9/11/2025 1:45:09 PM

Electropherogram Summary Continued ...

sample 0.8 brain female

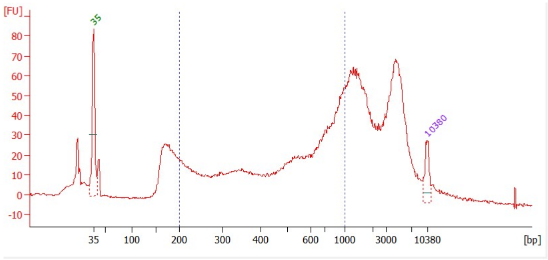

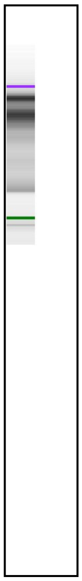

Overall Results for sample 1 : sample 0.8 brain female

Number of peaks found: 0
Corr. Area 1: 821.0
Noise: 0.3

Region table for sample 1 : sample 0.8 brain female

From [bp]	To [bp]	Corr. Area	% of Total	Average Size [bp]	Size distribution in CV [%]	Conc. [pg/µl]	Molarity [pmol/l]	Co lor
200	1,000	821.0	44	547	42.5	2,894.51	10,923.6	

© Copyright 2003 - 2018 Agilent, Inc.

Figure 2A_ Female_1x_Brain

Assay Class:	High Sensitivity DNA Assay	Created:	3/30/2022 3:45:08 PM
Data Path:	D:\...gh Sensitivity DNA Assay_DEDAE01941_2022-03-30_15-45-09.xad	Modified:	9/11/2025 1:45:09 PM

Electropherogram Summary Continued ...

sample 1 brain female

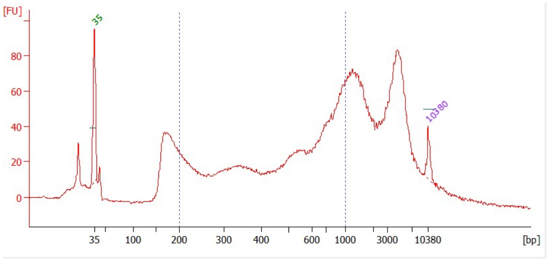

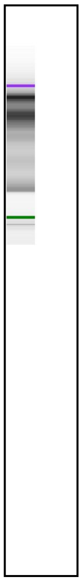

Overall Results for sample 2 : **sample 1 brain female**

Number of peaks found:	0	Corr. Area 1:	1,077.9
Noise:	0.4		

Region table for sample 2 : **sample 1 brain female**

From [bp]	To [bp]	Corr. Area	% of Total	Average Size [bp]	Size distribution in CV [%]	Conc. [pg/µl]	Molarity [pmol/l]	Co lor
200	1,000	1,077.9	45	540	42.6	6,126.00	23,306.5	

 © Copyright 2003 - 2018 Agilent, Inc.

Figure 2A_ Female_1.2x_Brain

Assay Class: High Sensitivity DNA Assay
Data Path: D:\...gh Sensitivity DNA Assay_DEDAE01941_2022-03-30_15-45-09.xad

Created: 3/30/2022 3:45:08 PM
Modified: 9/11/2025 1:45:09 PM

Electropherogram Summary Continued ...

sample 1.2 brain Female

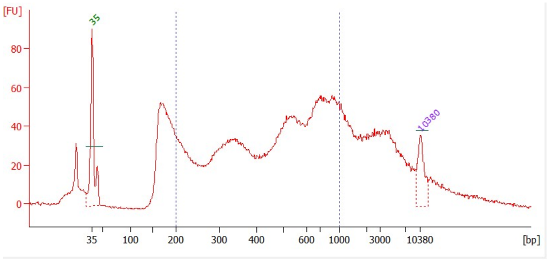

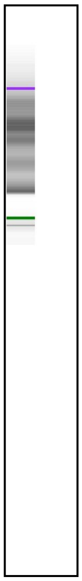

Overall Results for sample 3 : **<u>sample 1.2 brain Female</u>**

Number of peaks found: 0 Corr. Area 1: 1,495.1

Noise: 0.5

Region table for sample 3 : **<u>sample 1.2 brain Female</u>**

From [bp]	To [bp]	Corr. Area	% of Total	Average Size [bp]	Size distribution in CV [%]	Conc. [pg/µl]	Molarity [pmol/l]	Co lor
200	1,000	1,495.1	58	505	41.5	3,013.26	11,814.0	

© Copyright 2003 - 2018 Agilent, Inc.

Assay Class: High Sensitivity DNA Assay
Data Path: D:\...gh Sensitivity DNA Assay_DEDAE01941_2022-03-30_15-45-09.xad

Created: 3/30/2022 3:45:08 PM
Modified: 9/11/2025 1:45:09 PM

Electropherogram Summary Continued ...

sample 1.5 brain female

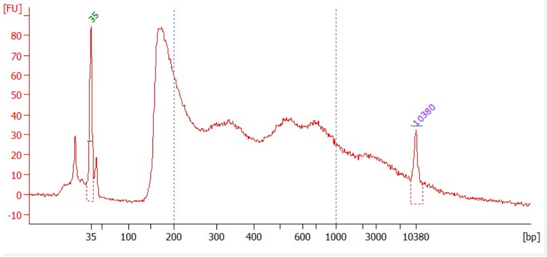

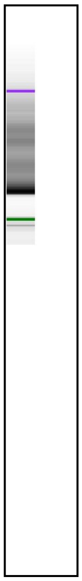

Overall Results for sample 4 : sample 1.5 brain female

Number of peaks found:	0	Corr. Area 1:	1,627.0
Noise:	0.6		

Region table for sample 4 : sample 1.5 brain female

From [bp]	To [bp]	Corr. Area	% of Total	Average Size [bp]	Size distribution in CV [%]	Conc. [pg/µl]	Molarity [pmol/l]	Color
200	1,000	1,627.0	60	443	44.0	3,980.89	17,609.4	

 © Copyright 2003 - 2018 Agilent, Inc.

Assay Class: High Sensitivity DNA Assay
Data Path: D:\...gh Sensitivity DNA Assay_DEDAE01941_2022-03-30_15-45-09.xad

Created: 3/30/2022 3:45:08 PM
Modified: 9/11/2025 1:45:09 PM

Electropherogram Summary Continued ...

sample 1.2 brain male

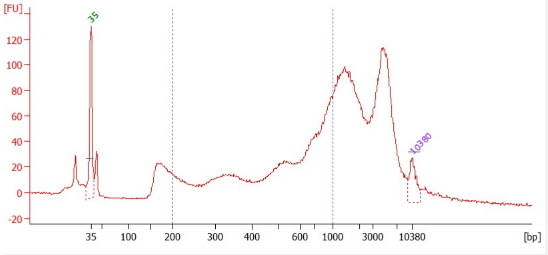

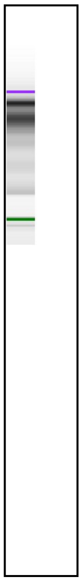

Overall Results for sample 5 : sample 1.2 brain male

Number of peaks found: 0
Noise: 0.5
Corr. Area 1: 981.6

Region table for sample 5 : sample 1.2 brain male

From [bp]	To [bp]	Corr. Area	% of Total	Average Size [bp]	Size distribution in CV [%]	Conc. [pg/µl]	Molarity [pmol/l]	Co lor
200	1,000	981.6	39	581	39.3	2,047.59	7,082.2	

© Copyright 2003 - 2018 Agilent, Inc.

Assay Class: High Sensitivity DNA Assay
Data Path: D:\...gh Sensitivity DNA Assay_DEDAE01941_2022-03-30_15-45-09.xad

Created: 3/30/2022 3:45:08 PM
Modified: 9/11/2025 1:45:09 PM

Electropherogram Summary Continued ...

sample 0.8 brain female 2

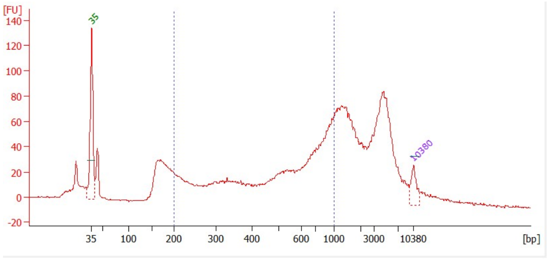

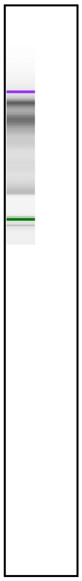

Overall Results for sample 6 : sample 0.8 brain female 2

Number of peaks found:	0	Corr. Area 1:	911.2
Noise:	0.4		

Region table for sample 6 : sample 0.8 brain female 2

From [bp]	To [bp]	Corr. Area	% of Total	Average Size [bp]	Size distribution in CV [%]	Conc. [pg/µl]	Molarity [pmol/l]	Co lor
200	1,000	911.2	42	556	41.7	2,488.21	9,203.5	

© Copyright 2003 - 2018 Agilent, Inc.

Assay Class: High Sensitivity DNA Assay
Data Path: D:\...gh Sensitivity DNA Assay_DEDAE01941_2022-03-30_15-45-09.xad

Created: 3/30/2022 3:45:08 PM
Modified: 9/11/2025 1:45:09 PM

Electropherogram Summary Continued ...

sample 1.0 brain female 2

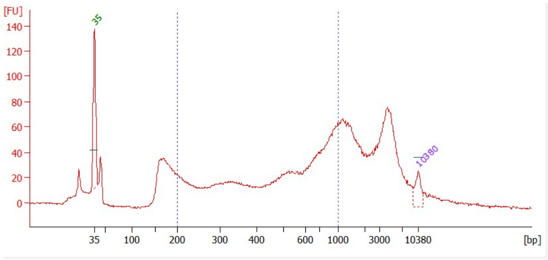

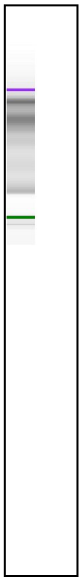

Overall Results for sample 7 : sample 1.0 brain female 2

Number of peaks found: 0

Corr. Area 1: 1,000.0

Noise: 0.5

Region table for sample 7 : sample 1.0 brain female 2

From [bp]	To [bp]	Corr. Area	% of Total	Average Size [bp]	Size distribution in CV [%]	Conc. [pg/µl]	Molarity [pmol/l]	Co lor
200	1,000	1,000.0	45	543	42.3	2,999.09	11,303.8	

© Copyright 2003 - 2018 Agilent, Inc.

Assay Class: High Sensitivity DNA Assay
Data Path: D:\...gh Sensitivity DNA Assay_DEDAE01941_2022-03-30_15-45-09.xad

Created: 3/30/2022 3:45:08 PM
Modified: 9/11/2025 1:45:09 PM

Electropherogram Summary Continued ...

sample 1.2 brain female 2

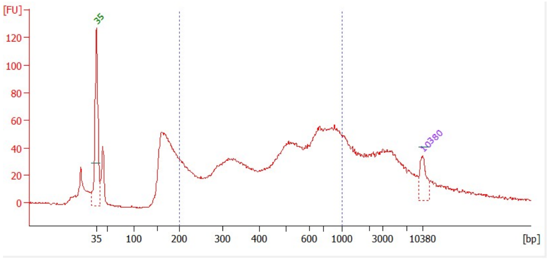

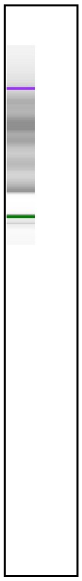

Overall Results for sample 8 : <u>sample 1.2 brain female 2</u>

Number of peaks found: 0

Corr. Area 1: 1,356.3

Noise: 0.5

Region table for sample 8 : <u>sample 1.2 brain female 2</u>

From [bp]	To [bp]	Corr. Area	% of Total	Average Size [bp]	Size distribution in CV [%]	Conc. [pg/µl]	Molarity [pmol/l]	Co lor
200	1,000	1,356.3	56	510	40.9	3,105.91	11,992.9	

© Copyright 2003 - 2018 Agilent, Inc.

Figure 2A_ Female_1.5x_Brain

Assay Class: High Sensitivity DNA Assay
Data Path: D:\...gh Sensitivity DNA Assay_DEDAE01941_2022-03-30_15-45-09.xad

Created: 3/30/2022 3:45:08 PM
Modified: 9/11/2025 1:45:09 PM

Electropherogram Summary Continued ...

sample 1.5 brain female 2

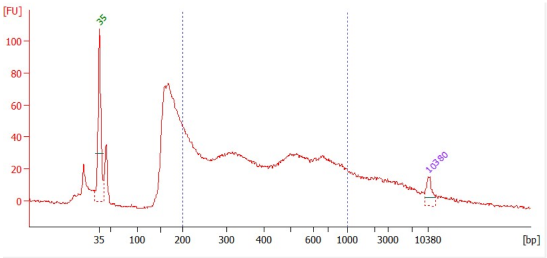

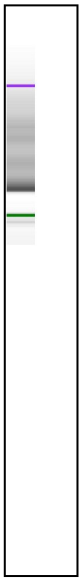

Overall Results for sample 9 : **sample 1.5 brain female 2**

Number of peaks found: 0
Noise: 0.7
Corr. Area 1: 1,254.8

Region table for sample 9 : **sample 1.5 brain female 2**

From [bp]	To [bp]	Corr. Area	% of Total	Average Size [bp]	Size distribution in CV [%]	Conc. [pg/µl]	Molarity [pmol/l]	Co lor
200	1,000	1,254.8	58	439	43.9	6,083.60	26,976.0	

 © Copyright 2003 - 2018 Agilent, Inc.

Assay Class: High Sensitivity DNA Assay
Data Path: D:\...gh Sensitivity DNA Assay_DEDAE01941_2022-03-30_15-45-09.xad

Created: 3/30/2022 3:45:08 PM
Modified: 9/11/2025 1:45:09 PM

Electropherogram Summary Continued ...

sample 1 brain male

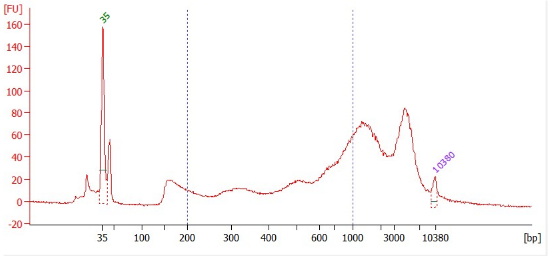

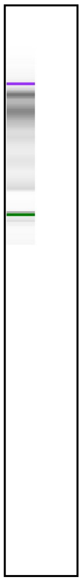

Overall Results for sample 10 : sample 1 brain male

Number of peaks found: 0 Corr. Area 1: 739.4

Noise: 0.5

Region table for sample 10 : sample 1 brain male

From [bp]	To [bp]	Corr. Area	% of Total	Average Size [bp]	Size distribution in CV [%]	Conc. [pg/µl]	Molarity [pmol/l]	Color
200	1,000	739.4	38	581	39.2	3,315.05	11,400.4	

 © Copyright 2003 - 2018 Agilent, Inc.

Assay Class:	High Sensitivity DNA Assay	Created:	3/30/2022 3:45:08 PM
Data Path:	D:\...gh Sensitivity DNA Assay_DEDAE01941_2022-03-30_15-45-09.xad	Modified:	9/11/2025 1:45:09 PM

Electropherogram Summary Continued ...

sample 1.2 brain male

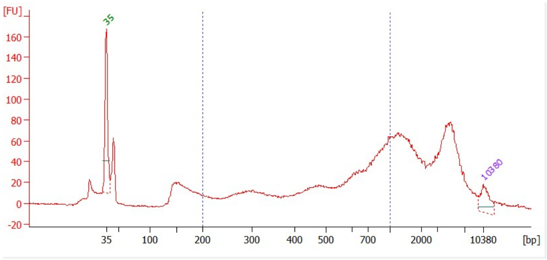

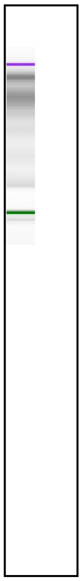

Overall Results for sample 11 : **<u>sample 1.2 brain male</u>**

Number of peaks found:	0	Corr. Area 1:	806.6
Noise:	0.6		

Region table for sample 11 : **<u>sample 1.2 brain male</u>**

From [bp]	To [bp]	Corr. Area	% of Total	Average Size [bp]	Size distribution in CV [%]	Conc. [pg/µl]	Molarity [pmol/l]	Color
200	1,000	806.6	40	590	38.3	1,813.99	6,118.6	

 © Copyright 2003 - 2018 Agilent, Inc.

Assay Class: High Sensitivity DNA Assay
Data Path: D:\...gh Sensitivity DNA Assay_DEDAE01941_2022-03-30_15-45-09.xad

Created: 3/30/2022 3:45:08 PM
Modified: 9/11/2025 1:45:09 PM

Gel Image

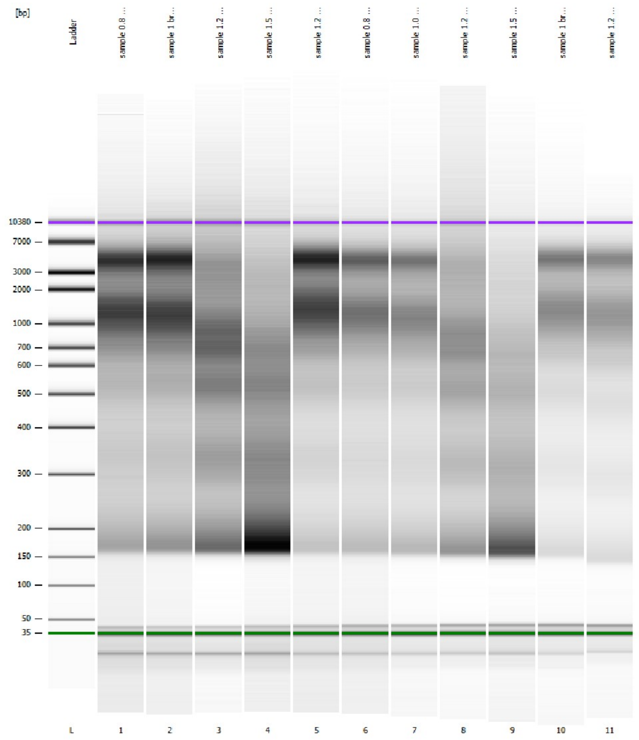

 © Copyright 2003 - 2018 Agilent, Inc.

Assay Class: High Sensitivity DNA Assay
Data Path: D:\...gh Sensitivity DNA Assay_DEDAE01941_2022-03-30_15-45-09.xad

Created: 3/30/2022 3:45:08 PM
Modified: 9/11/2025 1:45:09 PM

Run Logbook

Description	Number	Source	Category	Sub Category	Time	Time Zone	User	Host
Run ended on port 3 (Number of wells acquired: 12)		Instrument	Run		3/30/2022 4:26:27 PM	(GMT --04:00) Eastern Standard Time	harbison_lab	SBC-XL02213361
Run started on port 3 (File: C:\Program Files (x86)\Agilent\2100 bioanalyzer\2100 expert\Data\2022-03-30\2100 expert_High Sensitivity DNA Assay_DEDAE01941_2022-03-30_15-45-09.xad)		Instrument	Run		3/30/2022 3:45:13 PM	(GMT --04:00) Eastern Standard Time	harbison_lab	SBC-XL02213361
Product Number : G2939B		Instrument	Run		3/30/2022 3:45:13 PM	(GMT --04:00) Eastern Standard Time	harbison_lab	SBC-XL02213361
Name :		Instrument	Run		3/30/2022 3:45:13 PM	(GMT --04:00) Eastern Standard Time	harbison_lab	SBC-XL02213361
Vendor : Agilent Technologies		Instrument	Run		3/30/2022 3:45:13 PM	(GMT --04:00) Eastern Standard Time	harbison_lab	SBC-XL02213361
Serial# : DEDAE01941		Instrument	Run		3/30/2022 3:45:13 PM	(GMT --04:00) Eastern Standard Time	harbison_lab	SBC-XL02213361
Firmware : C.01.069		Instrument	Run		3/30/2022 3:45:13 PM	(GMT --04:00) Eastern Standard Time	harbison_lab	SBC-XL02213361
Cartridge : Electrode		Instrument	Run		3/30/2022 3:45:13 PM	(GMT --04:00) Eastern Standard Time	harbison_lab	SBC-XL02213361

 © Copyright 2003 - 2018 Agilent, Inc.

Assay Class: High Sensitivity DNA Assay
Data Path: D:\...gh Sensitivity DNA Assay_DEDAE01941_2022-03-31_15-09-11.xad

Created: 3/31/2022 3:09:10 PM
Modified: 9/11/2025 1:43:57 PM

Electrophoresis File Run Summary

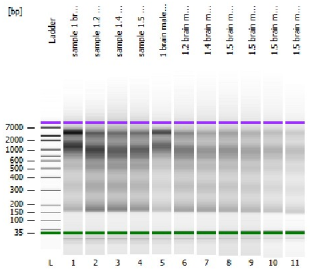

Instrument Information:

Instrument Name: DEDAE01941 Firmware: C.01.069

Serial#: DEDAE01941 Type: G2939B

Assay Information:

Assay Origin Path: C:\Program Files (x86)\Agilent\2100 bioanalyzer\2100 expert\assays\dsDNA\High Sensitivity DNA.xsy

Assay Class: High Sensitivity DNA Assay

Version: 1.03

Assay Comments: Copyright © 2003-2010 Agilent Technologies

Chip Information:

Chip Lot #:

Reagent Kit Lot #:

Chip Comments:

sample 1 brain male

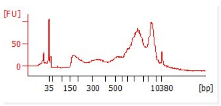

sample 1.2 brain male

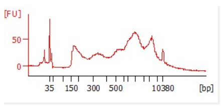

sample 1.4 brain male

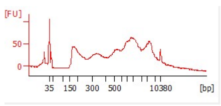

sample 1.5 brain male

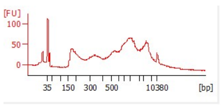

1 brain male replicate

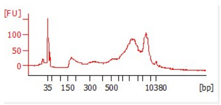

1.2 brain male replicate

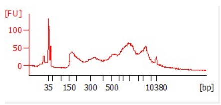

1.4 brain male replicate

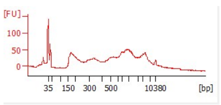

1.5 brain male replicate

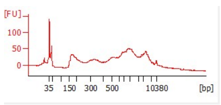

1.5 brain male replicate -2

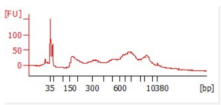

1.5 brain male replicate-2R

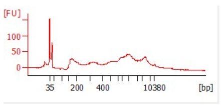

1.5 brain male replicate 2R

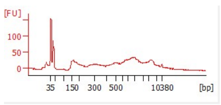

 © Copyright 2003 - 2018 Agilent, Inc.

Assay Class: High Sensitivity DNA Assay
Data Path: D:\...gh Sensitivity DNA Assay_DEDAE01941_2022-03-31_15-09-11.xad
Created: 3/31/2022 3:09:10 PM
Modified: 9/11/2025 1:43:57 PM

Electrophoresis File Run Summary (Chip Summary)

Sample Name	Sample Comment	Rest. Digest	Status	Observation	Result Label	Result Color
sample 1 brain male		☐	✔			
sample 1.2 brain male		☐	✔			
sample 1.4 brain male		☐	✔			
sample 1.5 brain male		☐	✔			
1 brain male replicate		☐	✔			
1.2 brain male replicate		☐	✔			
1.4 brain male replicate		☐	✔			
1.5 brain male replicate		☐	✔			
1.5 brain male replicate -2		☐	✔			
1.5 brain male replicate-2R		☐	✔			
1.5 brain male replicate 2R		☐	✔			
Ladder		☐	✔			

Chip Lot # **Reagent Kit Lot #**

Chip Comments :

 © Copyright 2003 - 2018 Agilent, Inc.

Assay Class: High Sensitivity DNA Assay
Data Path: D:\...gh Sensitivity DNA Assay_DEDAE01941_2022-03-31_15-09-11.xad
Created: 3/31/2022 3:09:10 PM
Modified: 9/11/2025 1:43:57 PM

Electrophoresis Assay Details

General Analysis Settings

Number of Available Sample and Ladder Wells (Max.) : 12
Minimum Visible Range [s] : 32
Maximum Visible Range [s] : 138
Start Analysis Time Range [s] : 33
End Analysis Time Range [s] : 137.5
Ladder Concentration [pg/µl] : 1950
Uses Standard Area for Ladder Fragments
Lower Marker Concentration [pg/µl] : 125
Upper Marker Concentration [pg/µl] : 75
Used Upper Marker for Quantitation
Standard Curve Fit is Point to Point
Show Data Aligned to Lower and Upper Marker

Integrator Settings

Integration Start Time [s] : 33.05
Integration End Time [s] : 137
Slope Threshold : 0.8
Height Threshold [FU] : 5
Area Threshold : 0.1
Width Threshold [s] : 0.6
Baseline Plateau [s] : 0.5

Filter Settings

Filter Width [s] : 0.5
Polynomial Order : 4

Ladder

Ladder Peak	Size	Area
1	35	160
2	50	210
3	100	208
4	150	221
5	200	242
6	300	270
7	400	305
8	500	306
9	600	336
10	700	321
11	1000	366
12	2000	413
13	3000	411
14	7000	400
15	10380	214

 © Copyright 2003 - 2018 Agilent, Inc.

Assay Class:	High Sensitivity DNA Assay	Created:	3/31/2022 3:09:10 PM
Data Path:	D:\...gh Sensitivity DNA Assay_DEDAE01941_2022-03-31_15-09-11.xad	Modified:	9/11/2025 1:43:57 PM

Electropherogram Summary

Ladder

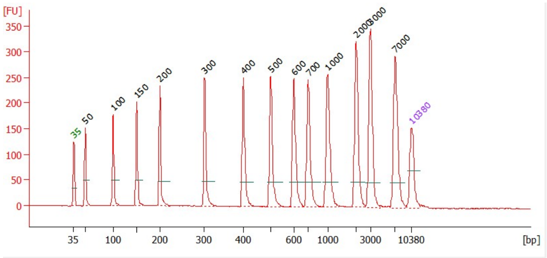

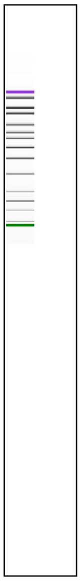

Overall Results for Ladder

Noise: 0.5

Peak table for Ladder

Peak		Size [bp]	Conc. [pg/µl]	Molarity [pmol/l]	Observations
2		50	150.00	4,545.5	Ladder Peak
3		100	150.00	2,272.7	Ladder Peak
4		150	150.00	1,515.2	Ladder Peak
5		200	150.00	1,136.4	Ladder Peak
6		300	150.00	757.6	Ladder Peak
7		400	150.00	568.2	Ladder Peak
8		500	150.00	454.5	Ladder Peak
9		600	150.00	378.8	Ladder Peak
10		700	150.00	324.7	Ladder Peak
11		1,000	150.00	227.3	Ladder Peak
12		2,000	150.00	113.6	Ladder Peak
13		3,000	150.00	75.8	Ladder Peak
14		7,000	150.00	32.5	Ladder Peak

 © Copyright 2003 - 2018 Agilent, Inc.

Assay Class: High Sensitivity DNA Assay
Data Path: D:\...gh Sensitivity DNA Assay_DEDAE01941_2022-03-31_15-09-11.xad

Created: 3/31/2022 3:09:10 PM
Modified: 9/11/2025 1:43:57 PM

Electropherogram Summary Continued ...

sample 1 brain male

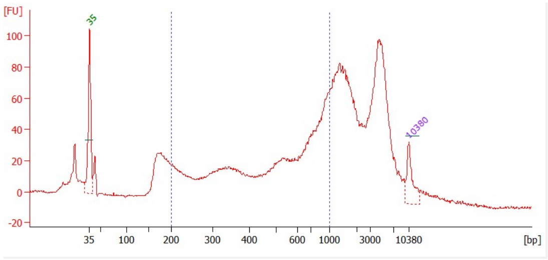

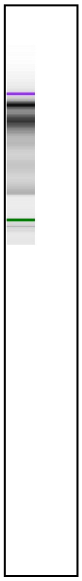

Overall Results for sample 1 : <u>sample 1 brain male</u>

Number of peaks found: 0

Corr. Area 1: 948.1

Noise: 0.5

Region table for sample 1 : <u>sample 1 brain male</u>

From [bp]	To [bp]	Corr. Area	% of Total	Average Size [bp]	Size distribution in CV [%]	Conc. [pg/µl]	Molarity [pmol/l]	Color
200	1,000	948.1	42	551	42.0	2,080.09	7,715.4	

 © Copyright 2003 - 2018 Agilent, Inc.

Figure 2A_Male_1.2x_Brain

Assay Class: High Sensitivity DNA Assay
Data Path: D:\...gh Sensitivity DNA Assay_DEDAE01941_2022-03-31_15-09-11.xad

Created: 3/31/2022 3:09:10 PM
Modified: 9/11/2025 1:43:57 PM

Electropherogram Summary Continued ...

sample 1.2 brain male

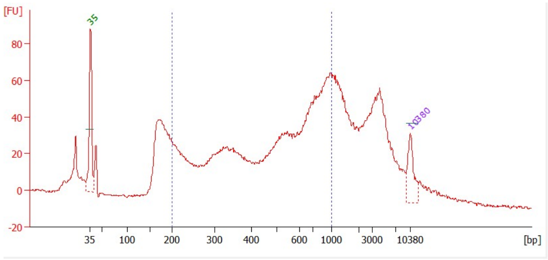

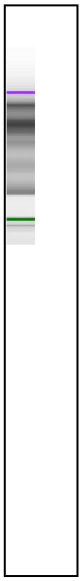

Overall Results for sample 2 : **sample 1.2 brain male**

Number of peaks found: 0 Corr. Area 1: 1,280.7

Noise: 0.5

Region table for sample 2 : **sample 1.2 brain male**

From [bp]	To [bp]	Corr. Area	% of Total	Average Size [bp]	Size distribution in CV [%]	Conc. [pg/µl]	Molarity [pmol/l]	Color
200	1,000	1,280.7	52	530	42.2	2,661.96	10,170.1	

 © Copyright 2003 - 2018 Agilent, Inc.

Assay Class: High Sensitivity DNA Assay
Data Path: D:\...gh Sensitivity DNA Assay_DEDAE01941_2022-03-31_15-09-11.xad

Created: 3/31/2022 3:09:10 PM
Modified: 9/11/2025 1:43:57 PM

Electropherogram Summary Continued ...

sample 1.4 brain male

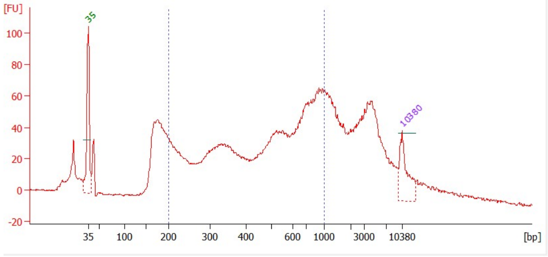

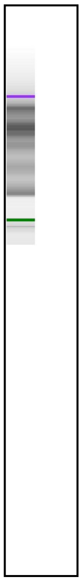

Overall Results for sample 3 : **sample 1.4 brain male**

Number of peaks found: 0 Corr. Area 1: 1,459.7

Noise: 0.5

Region table for sample 3 : **sample 1.4 brain male**

From [bp]	To [bp]	Corr. Area	% of Total	Average Size [bp]	Size distribution in CV [%]	Conc. [pg/µl]	Molarity [pmol/l]	Co lor
200	1,000	1,459.7	54	519	42.4	2,061.70	7,988.9	

 © Copyright 2003 - 2018 Agilent, Inc.

Figure 2A_Male_1.5x_Brain

Assay Class: High Sensitivity DNA Assay
Data Path: D:\...gh Sensitivity DNA Assay_DEDAE01941_2022-03-31_15-09-11.xad

Created: 3/31/2022 3:09:10 PM
Modified: 9/11/2025 1:43:57 PM

Electropherogram Summary Continued ...

sample 1.5 brain male

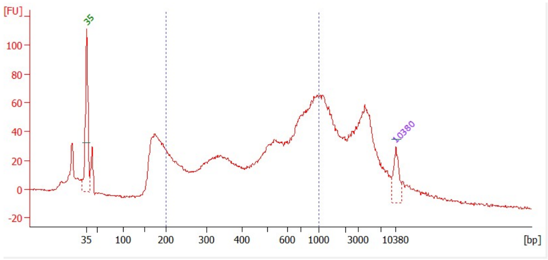

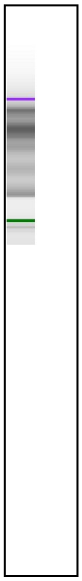

Overall Results for sample 4 : **sample 1.5 brain male**

Number of peaks found:	0	Corr. Area 1:	1,326.1
Noise:	0.7		

Region table for sample 4 : **sample 1.5 brain male**

From [bp]	To [bp]	Corr. Area	% of Total	Average Size [bp]	Size distribution in CV [%]	Conc. [pg/µl]	Molarity [pmol/l]	Co lor
200	1,000	1,326.1	52	534	41.6	3,095.18	11,684.9	

© Copyright 2003 - 2018 Agilent, Inc.

Assay Class:	High Sensitivity DNA Assay	Created:	3/31/2022 3:09:10 PM
Data Path:	D:\...gh Sensitivity DNA Assay_DEDAE01941_2022-03-31_15-09-11.xad	Modified:	9/11/2025 1:43:57 PM

Electropherogram Summary Continued ...

1 brain male replicate

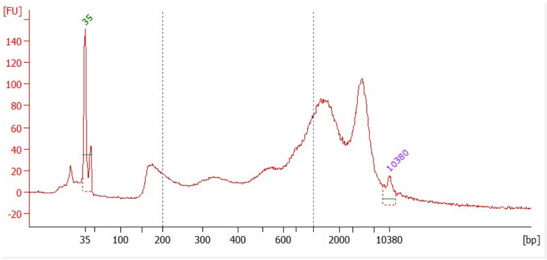

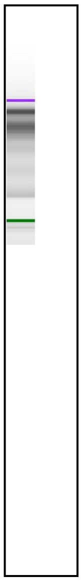

Overall Results for sample 5 : <u>1 brain male replicate</u>

Number of peaks found:	0	Corr. Area 1:	991.6
Noise:	0.6		

Region table for sample 5 : <u>1 brain male replicate</u>

From [bp]	To [bp]	Corr. Area	% of Total	Average Size [bp]	Size distribution in CV [%]	Conc. [pg/µl]	Molarity [pmol/l]	Co lor
200	1,000	991.6	41	566	40.6	2,318.31	8,311.1	

 © Copyright 2003 - 2018 Agilent, Inc.

Assay Class: High Sensitivity DNA Assay
Data Path: D:\...gh Sensitivity DNA Assay_DEDAE01941_2022-03-31_15-09-11.xad

Created: 3/31/2022 3:09:10 PM
Modified: 9/11/2025 1:43:57 PM

Electropherogram Summary Continued ...

1.2 brain male replicate

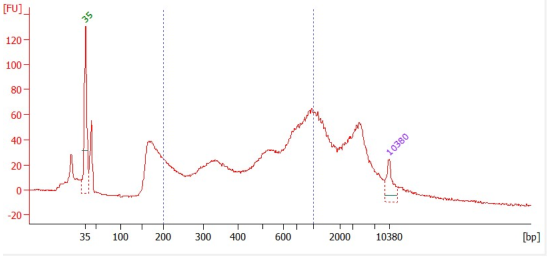

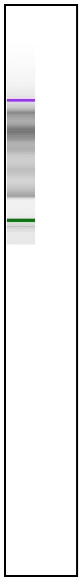

Overall Results for sample 6 : **1.2 brain male replicate**

Number of peaks found:	0	Corr. Area 1:	1,273.1
Noise:	0.5		

Region table for sample 6 : **1.2 brain male replicate**

From [bp]	To [bp]	Corr. Area	% of Total	Average Size [bp]	Size distribution in CV [%]	Conc. [pg/µl]	Molarity [pmol/l]	Co lor
200	1,000	1,273.1	51	536	41.4	2,823.43	10,590.2	

 © Copyright 2003 - 2018 Agilent, Inc.

Assay Class: High Sensitivity DNA Assay
Data Path: D:\...gh Sensitivity DNA Assay_DEDAE01941_2022-03-31_15-09-11.xad
Created: 3/31/2022 3:09:10 PM
Modified: 9/11/2025 1:43:57 PM

Electropherogram Summary Continued ...

1.4 brain male replicate

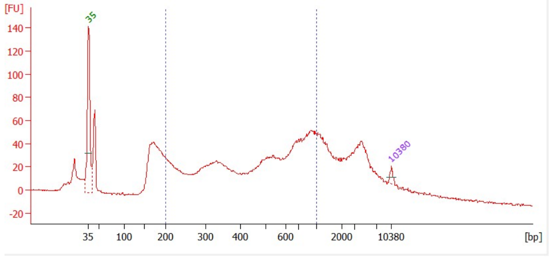

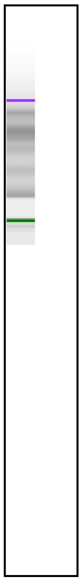

Overall Results for sample 7 : 1.4 brain male replicate

Number of peaks found: 0
Corr. Area 1: 1,232.8
Noise: 0.5

Region table for sample 7 : 1.4 brain male replicate

From [bp]	To [bp]	Corr. Area	% of Total	Average Size [bp]	Size distribution in CV [%]	Conc. [pg/µl]	Molarity [pmol/l]	Co lor
200	1,000	1,232.8	52	515	42.6	13,747.09	53,599.8	

© Copyright 2003 - 2018 Agilent, Inc.

Assay Class: High Sensitivity DNA Assay
Data Path: D:\...gh Sensitivity DNA Assay_DEDAE01941_2022-03-31_15-09-11.xad

Created: 3/31/2022 3:09:10 PM
Modified: 9/11/2025 1:43:57 PM

Electropherogram Summary Continued ...

1.5 brain male replicate

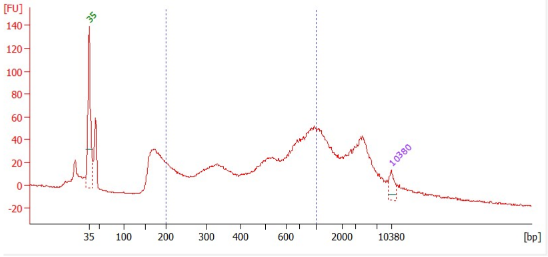

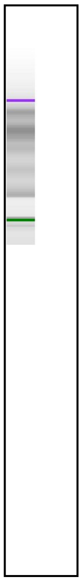

Overall Results for sample 8 : **1.5 brain male replicate**

Number of peaks found: 0

Corr. Area 1: 1,068.4

Noise: 0.6

Region table for sample 8 : **1.5 brain male replicate**

From [bp]	To [bp]	Corr. Area	% of Total	Average Size [bp]	Size distribution in CV [%]	Conc. [pg/µl]	Molarity [pmol/l]	Co lor
200	1,000	1,068.4	50	538	41.3	3,621.78	13,521.9	

 © Copyright 2003 - 2018 Agilent, Inc.

Assay Class: High Sensitivity DNA Assay
Data Path: D:\...gh Sensitivity DNA Assay_DEDAE01941_2022-03-31_15-09-11.xad

Created: 3/31/2022 3:09:10 PM
Modified: 9/11/2025 1:43:57 PM

Electropherogram Summary Continued ...

1.5 brain male replicate -2

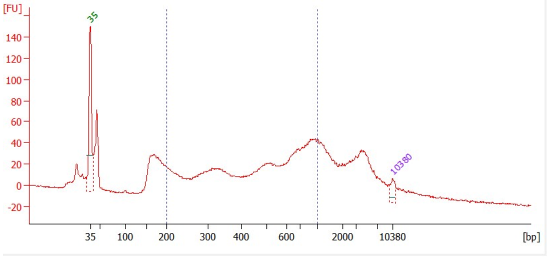

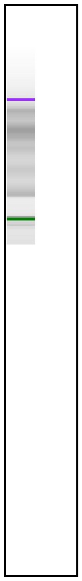

Overall Results for sample 9 : <u>1.5 brain male replicate -2</u>

Number of peaks found: 0

Corr. Area 1: 995.7

Noise: 0.8

Region table for sample 9 : <u>1.5 brain male replicate -2</u>

From [bp]	To [bp]	Corr. Area	% of Total	Average Size [bp]	Size distribution in CV [%]	Conc. [pg/µl]	Molarity [pmol/l]	Co lor
200	1,000	995.7	50	535	41.2	4,411.32	16,474.9	

 © Copyright 2003 - 2018 Agilent, Inc.

Assay Class: High Sensitivity DNA Assay
Data Path: D:\...gh Sensitivity DNA Assay_DEDAE01941_2022-03-31_15-09-11.xad

Created: 3/31/2022 3:09:10 PM
Modified: 9/11/2025 1:43:57 PM

Electropherogram Summary Continued ...

1.5 brain male replicate-2R

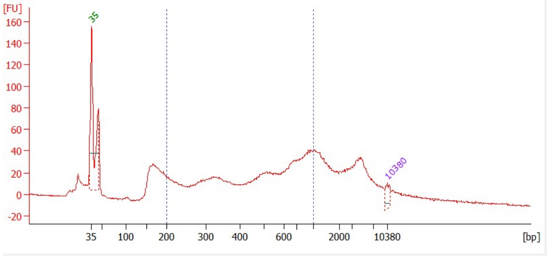

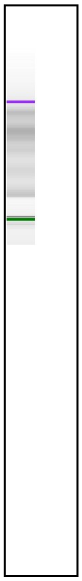

Overall Results for sample 10 : <u>1.5 brain male replicate-2R</u>

Number of peaks found: 0
Corr. Area 1: 838.4
Noise: 0.6

Region table for sample 10 : <u>1.5 brain male replicate-2R</u>

From [bp]	To [bp]	Corr. Area	% of Total	Average Size [bp]	Size distribution in CV [%]	Conc. [pg/µl]	Molarity [pmol/l]	Color
200	1,000	838.4	50	533	41.4	3,699.51	13,857.9	

 © Copyright 2003 - 2018 Agilent, Inc.

Assay Class: High Sensitivity DNA Assay
Data Path: D:\...gh Sensitivity DNA Assay_DEDAE01941_2022-03-31_15-09-11.xad

Created: 3/31/2022 3:09:10 PM
Modified: 9/11/2025 1:43:57 PM

Electropherogram Summary Continued ...

1.5 brain male replicate 2R

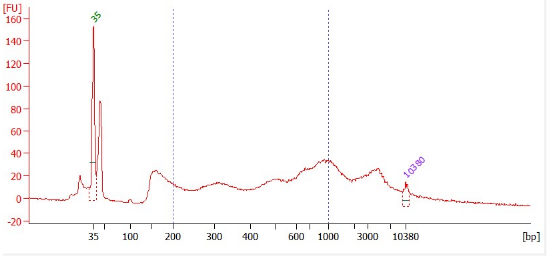

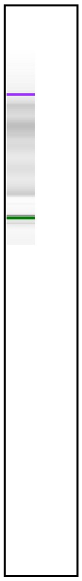

Overall Results for sample 11 : **1.5 brain male replicate 2R**

Number of peaks found: 0 Corr. Area 1: 708.0

Noise: 0.6

Region table for sample 11 : **1.5 brain male replicate 2R**

From [bp]	To [bp]	Corr. Area	% of Total	Average Size [bp]	Size distribution in CV [%]	Conc. [pg/µl]	Molarity [pmol/l]	Co lor
200	1,000	708.0	46	533	41.4	3,235.45	12,120.7	

 © Copyright 2003 - 2018 Agilent, Inc.

Assay Class: High Sensitivity DNA Assay
Data Path: D:\...gh Sensitivity DNA Assay_DEDAE01941_2022-03-31_15-09-11.xad

Created: 3/31/2022 3:09:10 PM
Modified: 9/11/2025 1:43:57 PM

Gel Image

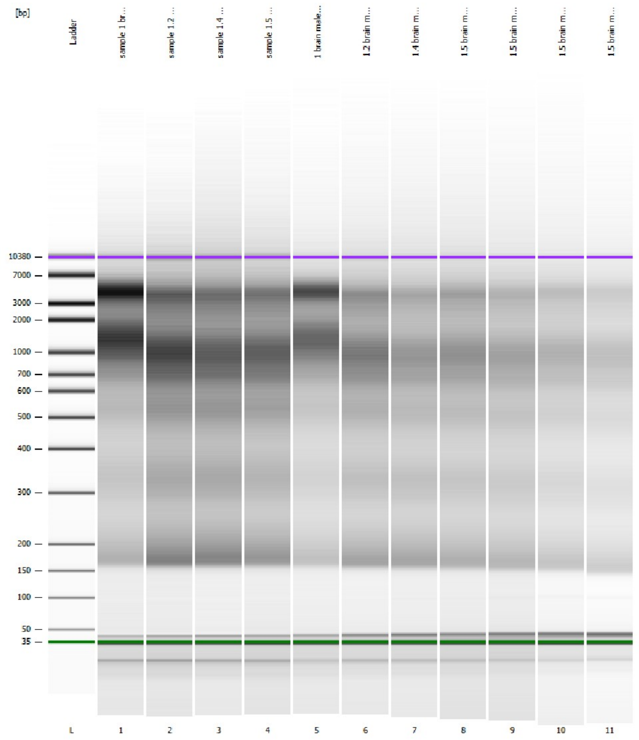

 © Copyright 2003 - 2018 Agilent, Inc.

Assay Class: High Sensitivity DNA Assay
Data Path: D:\...gh Sensitivity DNA Assay_DEDAE01941_2022-03-31_15-09-11.xad
Created: 3/31/2022 3:09:10 PM
Modified: 9/11/2025 1:43:57 PM

Run Logbook

Description	Number	Source	Category	Sub Category	Time	Time Zone	User	Host
Run ended on port 3 (Number of wells acquired: 12)		Instrument	Run		3/31/2022 3:50:30 PM	(GMT --04:00) Eastern Standard Time	harbison_lab	SBC-XL02213361
Run started on port 3 (File: C:\Program Files (x86)\Agilent\2100 bioanalyzer\2100 expert\Data\2022-03-31\2100 expert_High Sensitivity DNA Assay_DEDAE01941_2022-03-31_15-09-11.xad)		Instrument	Run		3/31/2022 3:09:16 PM	(GMT --04:00) Eastern Standard Time	harbison_lab	SBC-XL02213361
Product Number : G2939B		Instrument	Run		3/31/2022 3:09:16 PM	(GMT --04:00) Eastern Standard Time	harbison_lab	SBC-XL02213361
Name :		Instrument	Run		3/31/2022 3:09:16 PM	(GMT --04:00) Eastern Standard Time	harbison_lab	SBC-XL02213361
Vendor : Agilent Technologies		Instrument	Run		3/31/2022 3:09:16 PM	(GMT --04:00) Eastern Standard Time	harbison_lab	SBC-XL02213361
Serial# : DEDAE01941		Instrument	Run		3/31/2022 3:09:16 PM	(GMT --04:00) Eastern Standard Time	harbison_lab	SBC-XL02213361
Firmware : C.01.069		Instrument	Run		3/31/2022 3:09:16 PM	(GMT --04:00) Eastern Standard Time	harbison_lab	SBC-XL02213361
Cartridge : Electrode		Instrument	Run		3/31/2022 3:09:16 PM	(GMT --04:00) Eastern Standard Time	harbison_lab	SBC-XL02213361

 © Copyright 2003 - 2018 Agilent, Inc.

Assay Class:	High Sensitivity DNA Assay	Created:	4/3/2022 1:18:37 PM
Data Path:	D:\...gh Sensitivity DNA Assay_DEDAE01941_2022-04-03_13-18-38.xad	Modified:	9/10/2025 5:01:56 PM

Electrophoresis File Run Summary

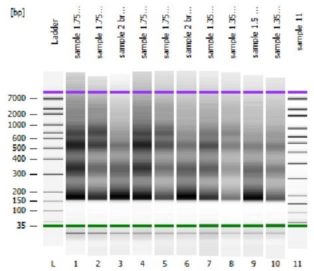

Instrument Information:

Instrument Name:	DEDAE01941	Firmware:	C.01.069
Serial#:	DEDAE01941	Type:	G2939B

Assay Information:

Assay Origin Path:	C:\Program Files (x86)\Agilent\2100 bioanalyzer\2100 expert\assays\dsDNA\High Sensitivity DNA.xsy
Assay Class:	High Sensitivity DNA Assay
Version:	1.03
Assay Comments:	Copyright © 2003-2010 Agilent Technologies

Chip Information:

Chip Lot #:

Reagent Kit Lot #:

Chip Comments:

sample 1.75 brain_Male

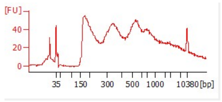

sample 1.75 brain_Male_replicate

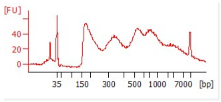

sample 2 brain_Male

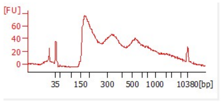

sample 1.75 brain male 2

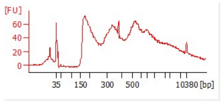

sample 1.75 brain male_replicate 2

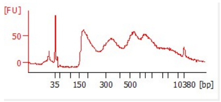

sample 2 brain male 2

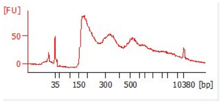

sample 1.35 brain_female

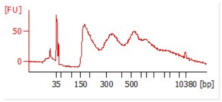

sample 1.35 brain_female-replicate

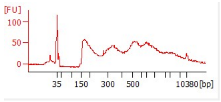

sample 1.5 brain female

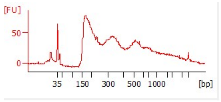

sample 1.35 brain_female2

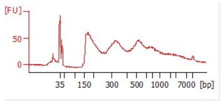

sample 11

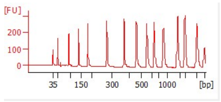

 © Copyright 2003 - 2018 Agilent, Inc.

Assay Class: High Sensitivity DNA Assay
Data Path: D:\...gh Sensitivity DNA Assay_DEDAE01941_2022-04-03_13-18-38.xad
Created: 4/3/2022 1:18:37 PM
Modified: 9/10/2025 5:01:56 PM

Electrophoresis File Run Summary (Chip Summary)

Sample Name	Sample Comment	Rest. Digest	Status	Observation	Result Label	Result Color
sample 1.75 brain_Male		☐	✓			
sample 1.75 brain_Male_replicate		☐	✓			
sample 2 brain_Male		☐	✓			
sample 1.75 brain male		☐	✓			
sample 1.75 brain male_replicate 2		☐	✓			
sample 2 brain male 2		☐	✓			
sample 1.35		☐	✓			
sample 1.35 brain_female-replicate		☐	✓			
sample 1.5 brain female		☐	✓			
sample 1.35 brain_female2		☐	✓			
sample 11		☐	✓			
Ladder		☐	✓			

Chip Lot # **Reagent Kit Lot #**

Chip Comments :

 © Copyright 2003 - 2018 Agilent, Inc.

Assay Class: High Sensitivity DNA Assay
Data Path: D:\...gh Sensitivity DNA Assay_DEDAE01941_2022-04-03_13-18-38.xad
Created: 4/3/2022 1:18:37 PM
Modified: 9/10/2025 5:01:56 PM

Electrophoresis Assay Details

General Analysis Settings

Number of Available Sample and Ladder Wells (Max.) : 12
Minimum Visible Range [s] : 32
Maximum Visible Range [s] : 138
Start Analysis Time Range [s] : 33
End Analysis Time Range [s] : 137.5
Ladder Concentration [pg/µl] : 1950
Uses Standard Area for Ladder Fragments
Lower Marker Concentration [pg/µl] : 125
Upper Marker Concentration [pg/µl] : 75
Used Upper Marker for Quantitation
Standard Curve Fit is Point to Point
Show Data Aligned to Lower and Upper Marker

Integrator Settings

Integration Start Time [s] : 33.05
Integration End Time [s] : 137
Slope Threshold : 0.8
Height Threshold [FU] : 5
Area Threshold : 0.1
Width Threshold [s] : 0.6
Baseline Plateau [s] : 0.5

Filter Settings

Filter Width [s] : 0.5
Polynomial Order : 4

Ladder

Ladder Peak	Size	Area
1	35	160
2	50	210
3	100	208
4	150	221
5	200	242
6	300	270
7	400	305
8	500	306
9	600	336
10	700	321
11	1000	366
12	2000	413
13	3000	411
14	7000	400
15	10380	214

© Copyright 2003 - 2018 Agilent, Inc.

Assay Class: High Sensitivity DNA Assay
Data Path: D:\...gh Sensitivity DNA Assay_DEDAE01941_2022-04-03_13-18-38.xad

Created: 4/3/2022 1:18:37 PM
Modified: 9/10/2025 5:01:56 PM

Electropherogram Summary

Ladder

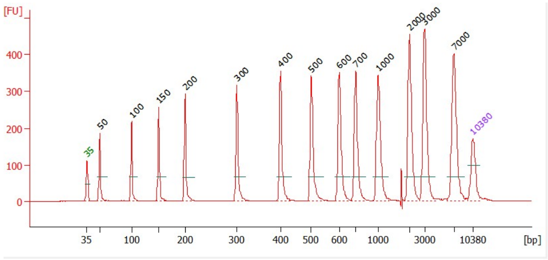

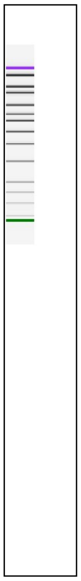

Overall Results for Ladder

Noise: 0.4

Peak table for Ladder

Peak		Size [bp]	Conc. [pg/µl]	Molarity [pmol/l]	Observations
2		50	150.00	4,545.5	Ladder Peak
3		100	150.00	2,272.7	Ladder Peak
4		150	150.00	1,515.2	Ladder Peak
5		200	150.00	1,136.4	Ladder Peak
6		300	150.00	757.6	Ladder Peak
7		400	150.00	568.2	Ladder Peak
8		500	150.00	454.5	Ladder Peak
9		600	150.00	378.8	Ladder Peak
10		700	150.00	324.7	Ladder Peak
11		1,000	150.00	227.3	Ladder Peak
12		2,000	150.00	113.6	Ladder Peak
13		3,000	150.00	75.8	Ladder Peak
14		7,000	150.00	32.5	Ladder Peak

 © Copyright 2003 - 2018 Agilent, Inc.

Figure 2B_Male_1.75x_Brain and Supplementary figure 2

Assay Class: High Sensitivity DNA Assay
Data Path: D:\...gh Sensitivity DNA Assay_DEDAE01941_2022-04-03_13-18-38.xad

Created: 4/3/2022 1:18:37 PM
Modified: 9/10/2025 5:01:56 PM

Electropherogram Summary Continued ...

sample 1.75 brain_Male

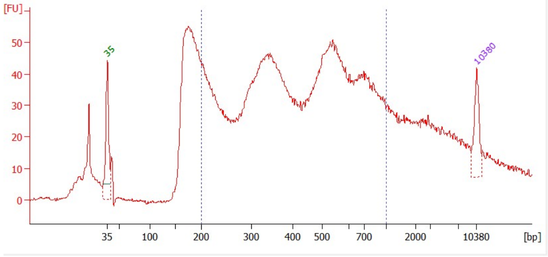

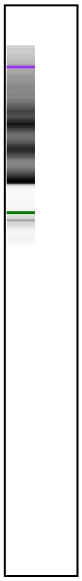

Overall Results for sample 1 : <u>sample 1.75 brain Male</u>

Number of peaks found: 0 Corr. Area 1: 1,451.8

Noise: 0.4

Region table for sample 1 : <u>sample 1.75 brain Male</u>

From [bp]	To [bp]	Corr. Area	% of Total	Average Size [bp]	Size distribution in CV [%]	Conc. [pg/µl]	Molarity [pmol/l]	Co lor
200	1,000	1,451.8	67	453	41.2	4,776.96	20,207.5	

 © Copyright 2003 - 2018 Agilent, Inc.

Assay Class: High Sensitivity DNA Assay
Data Path: D:\...gh Sensitivity DNA Assay_DEDAE01941_2022-04-03_13-18-38.xad

Created: 4/3/2022 1:18:37 PM
Modified: 9/10/2025 5:01:56 PM

Electropherogram Summary Continued ...

sample 1.75 brain_Male_replicate

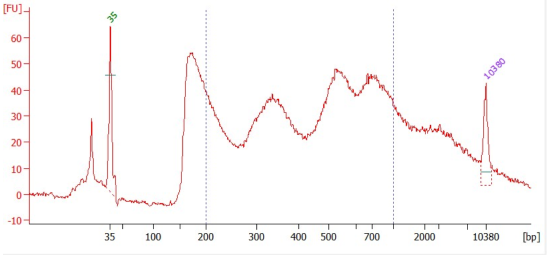

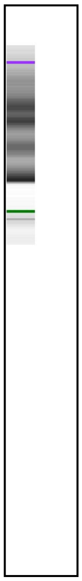

Overall Results for sample 2 : sample 1.75 brain_Male_replicate

Number of peaks found: 0

Corr. Area 1: 1,370.8

Noise: 0.5

Region table for sample 2 : sample 1.75 brain_Male_replicate

From [bp]	To [bp]	Corr. Area	% of Total	Average Size [bp]	Size distribution in CV [%]	Conc. [pg/µl]	Molarity [pmol/l]	Co lor
200	1,000	1,370.8	64	483	41.4	4,378.12	17,774.7	

© Copyright 2003 - 2018 Agilent, Inc.

Figure 2A_Male_2x_Brain

Assay Class: High Sensitivity DNA Assay | Created: 4/3/2022 1:18:37 PM
Data Path: D:\...gh Sensitivity DNA Assay_DEDAE01941_2022-04-03_13-18-38.xad | Modified: 9/10/2025 5:01:56 PM

Electropherogram Summary Continued ...

sample 2 brain_Male

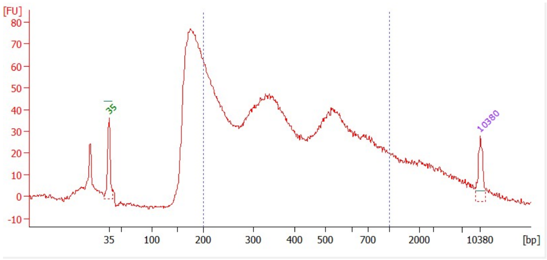

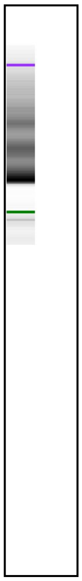

Overall Results for sample 3 : **sample 2 brain Male**

Number of peaks found: 0 Corr. Area 1: 1,681.5

Noise: 0.7

Region table for sample 3 : **sample 2 brain Male**

From [bp]	To [bp]	Corr. Area	% of Total	Average Size [bp]	Size distribution in CV [%]	Conc. [pg/µl]	Molarity [pmol/l]	Co lor
200	1,000	1,681.5	67	420	43.0	7,021.23	31,979.4	

 © Copyright 2003 - 2018 Agilent, Inc.

Assay Class: High Sensitivity DNA Assay
Data Path: D:\...gh Sensitivity DNA Assay_DEDAE01941_2022-04-03_13-18-38.xad
Created: 4/3/2022 1:18:37 PM
Modified: 9/10/2025 5:01:56 PM

Electropherogram Summary Continued ...

sample 1.75 brain male 2

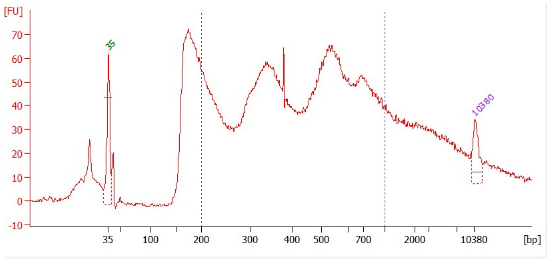

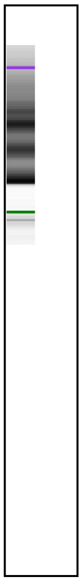

Overall Results for sample 4 : **sample 1.75 brain male 2**

Number of peaks found: 0
Corr. Area 1: 1,852.4
Noise: 0.9

Region table for sample 4 : **sample 1.75 brain male 2**

From [bp]	To [bp]	Corr. Area	% of Total	Average Size [bp]	Size distribution in CV [%]	Conc. [pg/µl]	Molarity [pmol/l]	Color
200	1,000	1,852.4	66	457	41.1	6,416.31	26,977.5	

© Copyright 2003 - 2018 Agilent, Inc.

Assay Class: High Sensitivity DNA Assay
Data Path: D:\...gh Sensitivity DNA Assay_DEDAE01941_2022-04-03_13-18-38.xad

Created: 4/3/2022 1:18:37 PM
Modified: 9/10/2025 5:01:56 PM

Electropherogram Summary Continued ...

sample 1.75 brain male_replicate 2

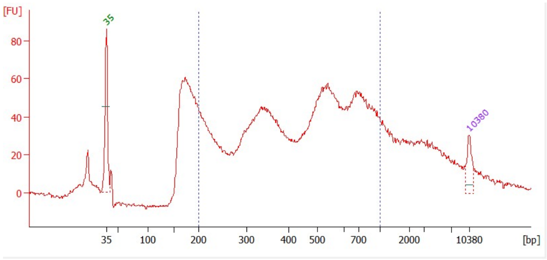

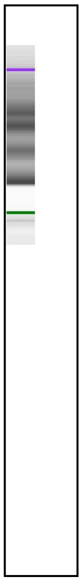

Overall Results for sample 5 : **sample 1.75 brain male_replicate 2**

Number of peaks found: 0

Corr. Area 1: 1,651.0

Noise: 0.7

Region table for sample 5 : **sample 1.75 brain male_replicate 2**

From [bp]	To [bp]	Corr. Area	% of Total	Average Size [bp]	Size distribution in CV [%]	Conc. [pg/µl]	Molarity [pmol/l]	Color
200	1,000	1,651.0	66	482	40.9	6,046.74	24,421.7	

© Copyright 2003 - 2018 Agilent, Inc.

Supplementary figure 2

Assay Class: High Sensitivity DNA Assay
Data Path: D:\...gh Sensitivity DNA Assay_DEDAE01941_2022-04-03_13-18-38.xad
Created: 4/3/2022 1:18:37 PM
Modified: 9/10/2025 5:01:56 PM

Electropherogram Summary Continued ...

sample 2 brain male 2

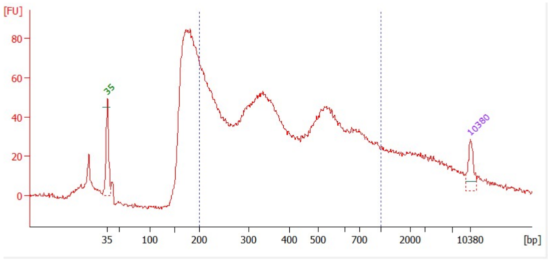

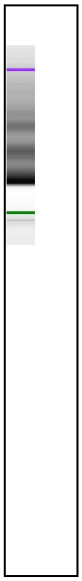

Overall Results for sample 6 : sample 2 brain male 2

Number of peaks found: 0
Corr. Area 1: 1,759.6
Noise: 0.8

Region table for sample 6 : sample 2 brain male 2

From [bp]	To [bp]	Corr. Area	% of Total	Average Size [bp]	Size distribution in CV [%]	Conc. [pg/µl]	Molarity [pmol/l]	Color
200	1,000	1,759.6	66	419	43.4	7,086.70	32,365.0	

© Copyright 2003 - 2018 Agilent, Inc.

Supplementary figure 2

Assay Class:	High Sensitivity DNA Assay	Created:	4/3/2022 1:18:37 PM
Data Path:	D:\...gh Sensitivity DNA Assay_DEDAE01941_2022-04-03_13-18-38.xad	Modified:	9/10/2025 5:01:56 PM

Electropherogram Summary Continued ...

sample 1.35 brain_female

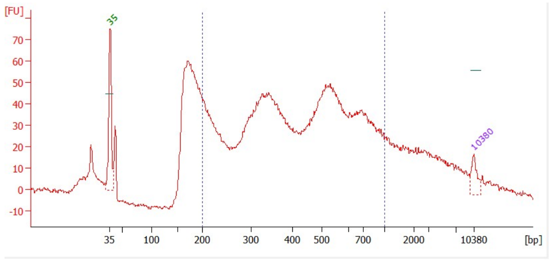

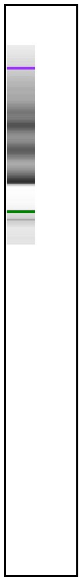

Overall Results for sample 7 : <u>sample 1.35 brain_female</u>

Number of peaks found:	0	Corr. Area 1:	1,570.6
Noise:	0.9		

Region table for sample 7 : <u>sample 1.35 brain_female</u>

From [bp]	To [bp]	Corr. Area	% of Total	Average Size [bp]	Size distribution in CV [%]	Conc. [pg/µl]	Molarity [pmol/l]	Co lor
200	1,000	1,570.6	65	456	40.7	8,181.67	34,209.2	

 © Copyright 2003 - 2018 Agilent, Inc.

Assay Class: High Sensitivity DNA Assay
Data Path: D:\...gh Sensitivity DNA Assay_DEDAE01941_2022-04-03_13-18-38.xad

Created: 4/3/2022 1:18:37 PM
Modified: 9/10/2025 5:01:56 PM

Electropherogram Summary Continued ...

sample 1.35 brain_female-replicate

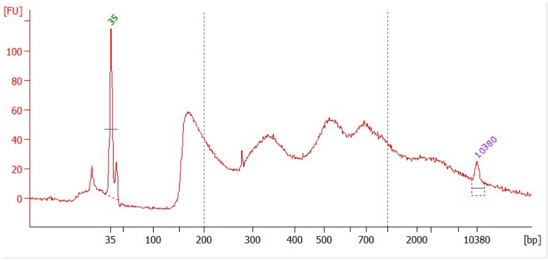

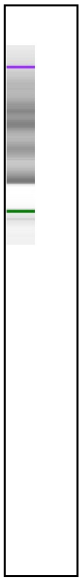

Overall Results for sample 8 : **sample 1.35 brain female-replicate**

Number of peaks found: 0

Noise: 0.6

Corr. Area 1: 1,568.4

Region table for sample 8 : **sample 1.35 brain female-replicate**

From [bp]	To [bp]	Corr. Area	% of Total	Average Size [bp]	Size distribution in CV [%]	Conc. [pg/µl]	Molarity [pmol/l]	Color
200	1,000	1,568.4	64	483	40.6	5,843.41	23,458.9	

© Copyright 2003 - 2018 Agilent, Inc.

Supplementary figure 2

Assay Class: High Sensitivity DNA Assay
Data Path: D:\...gh Sensitivity DNA Assay_DEDAE01941_2022-04-03_13-18-38.xad

Created: 4/3/2022 1:18:37 PM
Modified: 9/10/2025 5:01:56 PM

Electropherogram Summary Continued ...

sample 1.5 brain female

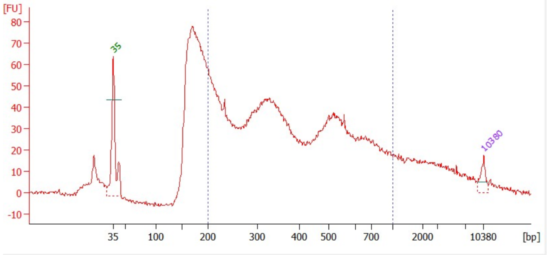

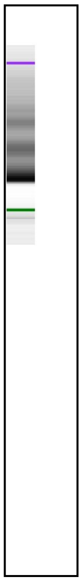

Overall Results for sample 9 : sample 1.5 brain female

Number of peaks found:	0	Corr. Area 1:	1,480.7
Noise:	0.7		

Region table for sample 9 : sample 1.5 brain female

From [bp]	To [bp]	Corr. Area	% of Total	Average Size [bp]	Size distribution in CV [%]	Conc. [pg/µl]	Molarity [pmol/l]	Color
200	1,000	1,480.7	64	413	43.1	10,672.19	49,119.1	

© Copyright 2003 - 2018 Agilent, Inc.

Figure 2B_Female_1.35x_Brain

Assay Class: High Sensitivity DNA Assay
Data Path: D:\...gh Sensitivity DNA Assay_DEDAE01941_2022-04-03_13-18-38.xad

Created: 4/3/2022 1:18:37 PM
Modified: 9/10/2025 5:01:56 PM

Electropherogram Summary Continued ...

sample 1.35 brain_female2

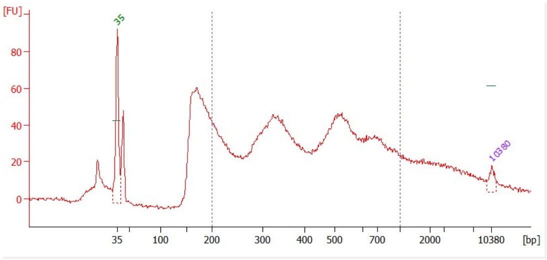

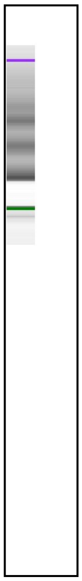

Overall Results for sample 10 : **sample 1.35 brain female2**

Number of peaks found: 0

Corr. Area 1: 1,348.0

Noise: 0.6

Region table for sample 10 : **sample 1.35 brain female2**

From [bp]	To [bp]	Corr. Area	% of Total	Average Size [bp]	Size distribution in CV [%]	Conc. [pg/µl]	Molarity [pmol/l]	Color
200	1,000	1,348.0	64	446	41.0	10,571.48	45,101.4	

© Copyright 2003 - 2018 Agilent, Inc.

Assay Class:	High Sensitivity DNA Assay	Created:	4/3/2022 1:18:37 PM
Data Path:	D:\...gh Sensitivity DNA Assay_DEDAE01941_2022-04-03_13-18-38.xad	Modified:	9/10/2025 5:01:56 PM

Electropherogram Summary Continued ...

sample 11

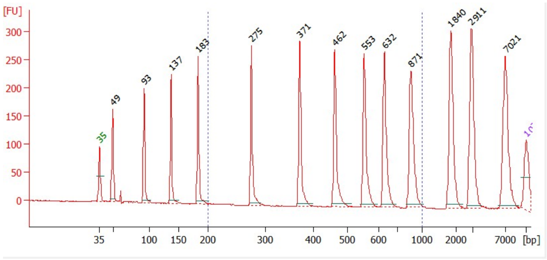

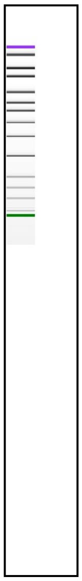

Overall Results for sample 11 : **sample 11**

Number of peaks found:	13	Corr. Area 1:	674.3
Noise:	0.6		

Peak table for sample 11 : **sample 11**

Peak	Size [bp]	Conc. [pg/µl]	Molarity [pmol/l]	Observations
2	49	211.20	6,557.2	
3	93	219.46	3,566.8	
4	137	218.87	2,424.0	
5	183	222.84	1,849.6	
6	275	213.72	1,176.2	
7	371	210.74	860.9	
8	462	211.19	693.1	
9	553	207.65	568.5	
10	632	204.96	491.6	
11	871	211.84	368.6	
12	1,840	200.50	165.1	
13	2,911	204.26	106.3	
14	7,021	201.04	43.4	

Region table for sample 11 : **sample 11**

From [bp]	To [bp]	Corr. Area	% of Total	Average Size [bp]	Size distribution in CV [%]	Conc. [pg/µl]	Molarity [pmol/l]	Co lor
200	1,000	674.3	44	547	35.7	679.72	2,325.9	

 © Copyright 2003 - 2018 Agilent, Inc.

Assay Class: High Sensitivity DNA Assay
Data Path: D:\...gh Sensitivity DNA Assay_DEDAE01941_2022-04-03_13-18-38.xad

Created: 4/3/2022 1:18:37 PM
Modified: 9/10/2025 5:01:56 PM

Gel Image

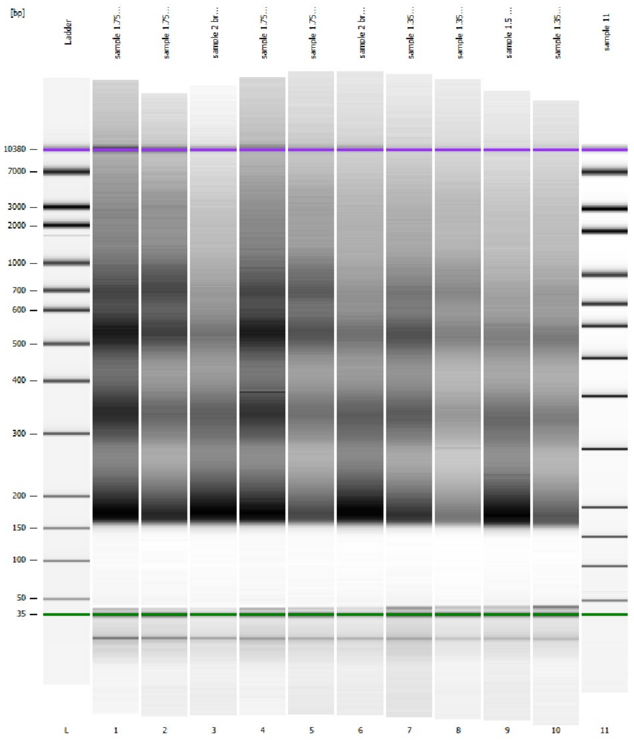

© Copyright 2003 - 2018 Agilent, Inc.

Assay Class: High Sensitivity DNA Assay
Data Path: D:\...gh Sensitivity DNA Assay_DEDAE01941_2022-04-03_13-18-38.xad

Created: 4/3/2022 1:18:37 PM
Modified: 9/10/2025 5:01:56 PM

Run Logbook

Description	Number	Source	Category	Sub Category	Time	Time Zone	User	Host
Run ended on port 3 (Number of wells acquired: 12)		Instrument	Run		4/3/2022 1:59:56 PM	(GMT --04:00) Eastern Standard Time	harbison_lab	SBC-XL02213361
Run started on port 3 (File: C:\Program Files (x86)\Agilent\2100 bioanalyzer\2100 expert\Data\2022-04-03\2100 expert_High Sensitivity DNA Assay_DEDAE01941_2022-04-03_13-18-38.xad)		Instrument	Run		4/3/2022 1:18:43 PM	(GMT --04:00) Eastern Standard Time	harbison_lab	SBC-XL02213361
Product Number : G2939B		Instrument	Run		4/3/2022 1:18:43 PM	(GMT --04:00) Eastern Standard Time	harbison_lab	SBC-XL02213361
Name :		Instrument	Run		4/3/2022 1:18:43 PM	(GMT --04:00) Eastern Standard Time	harbison_lab	SBC-XL02213361
Vendor : Agilent Technologies		Instrument	Run		4/3/2022 1:18:43 PM	(GMT --04:00) Eastern Standard Time	harbison_lab	SBC-XL02213361
Serial# : DEDAE01941		Instrument	Run		4/3/2022 1:18:43 PM	(GMT --04:00) Eastern Standard Time	harbison_lab	SBC-XL02213361
Firmware : C.01.069		Instrument	Run		4/3/2022 1:18:43 PM	(GMT --04:00) Eastern Standard Time	harbison_lab	SBC-XL02213361
Cartridge : Electrode		Instrument	Run		4/3/2022 1:18:42 PM	(GMT --04:00) Eastern Standard Time	harbison_lab	SBC-XL02213361

© Copyright 2003 - 2018 Agilent, Inc.
